# Dietary Fat Intake Attenuates Vitamin A Deficiency-Associated Elastic Fiber Remodeling and Lipid Reduction in the Alveolar Niche in Mice

**DOI:** 10.1016/j.tjnut.2025.07.010

**Published:** 2025-07-17

**Authors:** Lisa-Marie Hoy, Tabea Meier, Natascha Mierswa, Melanie Bornemann, Lea Naasner, Heike Bähre, Natali Froese, Christian Riehle, Christian Mühlfeld, Julia Schipke

**Affiliations:** 1 Hannover Medical School, Institute of Functional and Applied Anatomy, Hannover, Germany; 2 Hannover Medical School, Department of Cardiology and Angiology, Hannover, Germany; 3 Research Core Unit Metabolomics, Institute of Pharmacology, Hannover Medical School, Hannover, Germany; 4 German Center for Lung Research (DZL), Biomedical Research in Endstage and Obstructive Lung Disease Hannover (BREATH), Hannover, Germany

**Keywords:** vitamin A deficiency, obesity, alveolar region, lipid depletion, elastic fiber remodeling, fibrillin-1, alveolar epithelial type 2 cells, LRAT-Knockout

## Abstract

**Background:**

Vitamin A deficiency (VAD) and obesity are widespread nutrition-related health conditions that are independently associated with pulmonary remodeling processes linked to lung function decline and respiratory diseases.

**Objectives:**

This study tested the hypothesis that VAD-related pulmonary alterations are aggravated by diet-induced obesity.

**Methods:**

Eight-week-old C57BL/6J/129Sv mice with a deletion of lecithin-retinol-acyltransferase (*Lrat−*; impaired vitamin A storage) were fed vitamin A deficient control diet (CD, *n =* 13) or high-fat diet (HFD, *n =* 15) to induce VAD in lean (CD-VAD, *n =* 13) or obese (HFD-VAD, *n =* 13) mice. Wild-type mice receiving vitamin A-containing CD or HFD served as controls. After 20 wk, lungs were subjected to structural and molecular analyses by stereology, western blot, and high-pressure liquid chromatography-mass spectrometry. Statistics used were 2-way analysis of variance.

**Results:**

Pulmonary vitamin A reserves were efficiently depleted in CD-VAD and HFD-VAD (*P <* 0.001 compared with controls). In CD-VAD, 76% of pulmonary elastic fibers appeared densely packed (CD: 53%, *P <* 0.01), and expression of fibrillin was 110% higher compared with CD (*P <* 0.01), indicating a higher septal microfibril content. Elastin expression was slightly reduced in HFD-groups (HFD: 14%, HFD-VAD: 16% of respective controls, both *P <* 0.05), whereas neither diet nor VAD affected expression levels of collagen I or III. Lipid droplet volumes decreased by 32% in septal fibroblasts (*P <* 0.05) and by 53% in alveolar epithelial type 2 (AE2) cells in CD-VAD, compared with CD. HFD alone led to a 20% reduction in lung airspace volume, a 13% decrease in septal surface area, and a 15% reduction in AE2 cell numbers compared with CD. These VAD- and obesity-related changes were alleviated or absent in HFD-VAD.

**Conclusions:**

VAD-induced elastic fiber remodeling and lipid droplet reduction in the alveolar region of lean mice, whereas HFD resulted in smaller lungs containing less AE2 cells. Both VAD- and obesity-related effects were attenuated in HFD-VAD, indicating mutually mitigating effects.

## Graphical abstract

**Figure:**
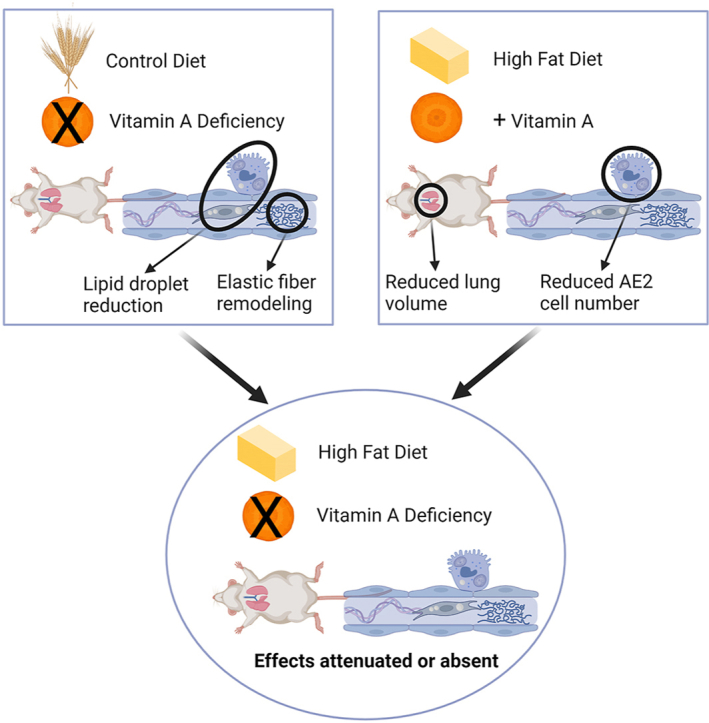

## Introduction

Vitamin A is a collective term for different retinoids present in the body, such as retinol, retinal, retinoic acid, and retinyl ester. These essential, fat-soluble micronutrients must be consumed with the diet, either as preformed vitamin A from animal sources or as provitamin A from plant-based foods [1]. Retinoids have an unsaturated, hydrophobic isoprenoid side chain and a functional group. This allows them to interact with nuclear receptors, such as the retinoic-acid-receptor, the retinoid-x-receptor, or the peroxisome proliferator-activated receptor [1,2]. Retinoic acid modulates the expression of >500 target genes [[3], [4], [5]]. This explains its pleiotropic effects on lipid metabolism, energy homeostasis, immune function, vision, antioxidation, cell regeneration, and on fetal organ development, particularly lung formation and maturation [[6], [7], [8], [9]].

After the postprandial uptake of retinol into the enterocytes, it is converted to retinyl ester by lecithin-retinol-acyltransferase (LRAT). The esterified form is packaged into chylomicrons and released into the portal circulation [10]. Hepatocytes take up the chylomicrons from the blood and hydrolyze the retinyl esters to retinol. Retinol is then bound to the retinol-binding protein 4 (RBP4) and delivered to the hepatic stellate cells for storage or released into the bloodstream to reach the peripheral organs. Hepatic retinyl esters represent ∼50%–80% of the body's vitamin A storage [11]. Additionally, vitamin A is stored in extrahepatic cells, including adipocytes and pulmonary cells within the alveolar septa [3,11,12]. Lung retinyl ester concentrations were ∼60 nmol/g, corresponding to ∼40% of the hepatic retinyl ester concentrations [3].

Adipose tissue not only stores vitamin A but also has an active vitamin A metabolism. There are indications that retinoids and retinoic acid receptors are crucial in controlling obesity, energy consumption, and insulin sensitivity [13]. When required, the vitamin A reserves are mobilized, releasing the previously hydrolyzed retinol into the circulation. To reach peripheral organs, retinol binds to the RBP4 [10,13]. RBP4, mainly synthesized by the liver and adipose tissue, is also referred to as an adipokine. In obesity, elevated levels of RBP4 have been linked to the onset of metabolic diseases, including obesity, type 2 diabetes mellitus, and the metabolic syndrome [14,15].

Vitamin A deficiency (VAD) is 1 of the 4 most common nutritional deficiencies worldwide and is linked to visual impairment and an increased risk of infectious diseases in childhood [16,17]. Moreover, the lung also appears to be affected by VAD. Baybutt et al. [18] observed emphysematous changes in the lungs of VAD rats, including increased volumes of distal air spaces, which were associated with partial or complete destruction of the alveolar septa. Furthermore, VAD induces changes in the composition and the distribution of proteins in the extracellular matrix (ECM) and alveolar basement membrane within alveolar septa [6]. Pulmonary collagens possess high tensile strength and are essential for maintaining the structural integrity of the lung [19]. Collagen I is increased in the lungs of rats with VAD, and deposited ectopically in the alveolar basement membrane, doubling its thickness [20]. Patients with fibrotic diseases typically also show these morphological changes, suggesting that VAD may contribute to the development and progression of pulmonary fibrosis [21,22]. In contrast, a decrease in the amount of septal collagen fibers has also been observed in VAD mice [23]. Moreover, VAD has been associated with a decrease in pulmonary elastic fibers and elastin in rodent models [18,24,25]. Additionally, VAD impairs the function of alveolar epithelial type 2 (AE2) cells, leading to diminished epithelial regeneration and surfactant dysfunction [18,26]. AE2 cells are critical for lung function, as they serve as progenitors for AE1 cells covering >95% of the alveolar surface area and produce the surfactant, preventing alveolar collapse [27,28]. Moreover, VAD impairs pulmonary lipid metabolism in rats, leading to reduced surfactant synthesis [18]. Overall, these processes are associated with epithelial apoptosis and the development of acute lung injury as well as acute respiratory distress syndrome [26,28,29].

Another systemic condition affecting the lung is obesity, leading to morphological changes in the alveolar septa. In mice, diet-induced obesity induces a thickening of the septal endothelium and the air–blood barrier, which is associated with hyperventilation [30,31]. Moreover, lipid accumulation in septal fibroblasts and epithelial cells has been observed in obese rodents [32,33]. The diet-induced increase of lipid droplets in AE2 cells is linked to alterations in surfactant lipid composition and function in obese mice [33]. Obesity-related lipid deposition in the lung is associated with lipotoxic effects, leading to endoplasmic reticulum stress and apoptosis of alveolar epithelial cells [34]. These findings are also frequently observed in patients with pulmonary fibrosis [35].

The prevalence of both, obesity and VAD, are public health concerns that increasingly affect the same population groups [16,[36], [37], [38]]. Furthermore, both conditions are independently associated with pulmonary remodeling processes. However, it remains elusive whether there is an amplifying effect of obesity and VAD, particularly with regard to the effects on the alveolar region. Thus, this study tested the hypothesis that VAD-induced alterations of the septal ECM, lipid distribution, and AE2 cells are aggravated by diet-induced obesity.

## Methods

### Mice studies

**FIGURE 1:**
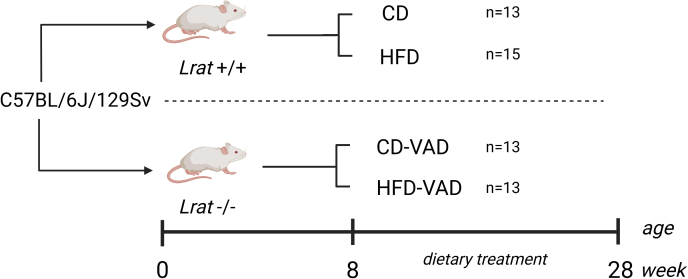
Study design. Male C57BL/6J/129Sv mice of 2 genotypes were used: wild-type (*Lrat*+/+) and LRAT-knockout (*Lrat−*/*−*). Dietary treatment started at 8-wk of age. Wild-type mice were fed either a control diet (CD, *n =* 13) or a high-fat diet (HFD, *n =* 15), and *Lrat−*/*−* mice were fed a vitamin A deficient control diet (CD-VAD, *n =* 13) or a vitamin A deficient high-fat diet (HFD-VAD, *n =* 13). After 20 wk, lungs were harvested and further analyzed. LRAT, lecithin-retinol-acyltransferase; VAD, vitamin A deficiency.

Male C57BL/6J/129Sv mice with a deletion of *Lrat−/−* gene (impaired vitamin A storage) were purchased from Jackson Laboratories (Bar Harbor). Genotyping was performed, and results were published previously [8,39]. Mice were randomly allocated to the different diet groups. To induce VAD in lean or obese mice, *Lrat−*/*−* mice were fed a vitamin A deficient control diet, with 13% kcal from fat (CD-VAD, *n =* 13) or a vitamin A deficient high-fat diet, with 60% kcal from fat (HFD-VAD, *n =* 13). Wild-type mice (*Lrat*+/+) receiving control or high-fat diet served as controls (CD, *n =* 13; HFD, *n =* 15). This group size was based on a F-test, 1-way analysis of variance (ANOVA), a priori power analysis with a critical significance level of 0.05, a power of 0.8, and an effect size of 0.5. All diets were purchased from Altromin, and the composition is shown in Supplemental Table 1. Dietary treatment was initiated at 8 wk of age for a total duration of 20-wk (Figure 1). Mice were maintained on a 12-h-light/dark cycle and housed with ad libitum access to food and water. All experiments were approved by the local state authorities (Lower Saxony State Office for Consumer Protection and Food Safety, Protocol No. 17/2702). After 20 wk of dietary treatment, mice were killed by cervical dislocation under deep anesthesia, lungs were isolated and further processed. For analysis of vitamin A derivatives, organs were harvested under dim red light. Right lung lobes and epididymal white adipose tissue depots were snap frozen and stored at −80°C for molecular analyses. The left lung lobe was fixed via tracheal instillation at a hydrostatic pressure of 20 cm H_2_O with 1.5% paraformaldehyde (Sigma-Aldrich) and 1.5% glutaraldehyde (Merck) in 0.15 M 4-(2-hydroxyethyl)-1-piperazineethane sulfonic acid buffer at pH 7.35 (Sigma-Aldrich), and kept in the fixative solution for ≥24 h.

### Structural analysis by design-based stereology

Structural analyses of the lung were performed by design-based stereology, according to the recommendations of the American Thoracic Society and the European Respiratory Society [40]. The volume of the left lung was determined by fluid displacement according to Archimedes' principle [41]. Left lung lobes were subjected to systematic uniform random sampling (SURS) and tissue slices were randomly assigned to light microscopy (LM) or transmission electron microscopy (TEM) analysis [42]. The analyst was blinded for experimental groups.

The samples for LM analysis were embedded in glycol methacrylate (Technovit 7100; Heraeus Kulzer) according to the manufacturer's instructions. Sections 1.5 μm thick were cut; the first and third sections were mounted on slides and stained with toluidine blue (Merck). Sections were digitized with a slide scanner (AxioScan.Z1, Zeiss). For analysis of the lung parenchyma and alveolar septa, a primary magnification of 20×, and for analysis of AE2 cell numbers, a primary magnification of 40× was used. Stereological analyses were performed using the newCast software (Visiopharm). Fields of view were generated using the SURS method [42]. The number of images analyzed was based on the aimed number of counting events (100–200) for the different parameters (∼60–80 fields of view per section). Parameters analyzed at the LM level were volumes of lung parenchyma, alveolar septa, and alveolar/ductal airspace, the alveolar surface area, the septal thickness, the number of AE2 cells, and the number-weighted mean volume of AE2 cells. The mean linear intercept was calculated from the assessed parameters. Details on analyses and calculations are described previously [40,42].

The samples for TEM analysis were embedded in epoxy resin (Epon, Serva) as described previously [43]. Sections 60 nm thin were mounted on copper grids, contrasted with uranyl acetate, and 3 randomly selected sections per mouse were included in the analysis. For the analysis of AE2 cell composition, 30 random fields of view per section were recorded with a digital camera (Veleta; Olympus Soft Imaging Solutions) with a Morgagni 268 electron microscope (FEI) at a primary magnification of 7100×. For all other parameters, 40 random fields of view per section were sampled at a primary magnification of 14,000×. For analysis, the STEPanizer stereology tool was used [44]. Parameters analyzed at the TEM-level were volumes of epithelium, endothelium, interstitium, and its components (interstitial cells, elastic, and collagen fibers), lipid droplet volumes within septal cells, and lamellar body volumes within AE2 cells. Further details on counting methods are described in the literature [40,42].

Furthermore, the structural appearance of elastic fibers was assessed by a semiquantitative scoring, as described previously [45].

### Measurements of lung and adipose tissue retinoid levels

Tissue retinoid concentrations were measured by HPLC in combination with mass spectrometry (MS). Approximately, 70 mg lung tissue and 50 mg epididymal adipose tissue were transferred under dim red light to dark brown FastPrep tubes (Lysing Matrix A). Then, 600 μL of ice-cold extraction solvent (2/2/1 (vol/vol) acetonitrile/methanol/water) containing 50 nM d5-*all-trans*-retinol (Sigma-Aldrich; 97%), 200 nM-d4-*all-trans*-retinyl palmitate (Sigma-Aldrich; 98%), and 25 nM d5-*all-trans*-retinoic acid (Sigma-Aldrich; 97.5%) as internal standards were added.

Homogenization was performed using the FastPrep-24 device (MP Biomedicals). After centrifugation, 100 μL supernatants were transferred to dark brown analysis vials, which were then subjected to HPLC-MS (LC-MS/MS API4000, Sciex). An electrospray ionization source (ESI) operating in positive ionization mode was connected to the HPLC-MS. ESI parameters were as follows: Ion spray voltage, 4500 V; curtain gas (CUR), 30 psi; collision gas (CAD), 9; temperature, 400°C; gas 1, 60 psi; and gas 2, 75 psi. Multiple reaction monitoring transitions were optimized for selective reaction monitoring, and the most intense transitions for *all-trans*-retinoic acid, *all-trans*-retinyl palmitate, and *all-trans*-retinol were identified as 300→123, 269→77, and 269→77, respectively. Levels of *all-trans-*retinol, *all-trans-*retinyl palmitate, and *all-trans-*retinoic acid were quantified using d5-*all-trans-*retinol (mass transition 274→142), d4-*all-trans-*retinyl palmitate (mass transition 273→93), and d5-*all-trans-*retinoic acid (mass transition 290→161) as internal standards. Quantification was performed using the ratio of the analyte peak area and the internal standard. Concentrations of metabolites were calculated using a calibration curve generated with quadratic regression and a weighting factor of 1/×, as implemented in Analyst 1.7 software (Sciex).

### Protein isolation and immunoblotting

Approximately 20 mg lung tissue was homogenized using a Tissue Lyser (Qiagen). Lysates were sonicated, centrifuged, and the protein content was determined using the bicinchoninic acid assay (PierceTM BCA Kit, Thermo Fisher Scientific). A Multiskan SkyHigh Microplate Spectrophotometer (Thermo Fisher Scientific) was used to measure the absorbances at a wavelength of 592 nm with Skanlt Software Microplate Readers (Thermo Fisher Scientific).

A total of 20–30 μg proteins per lane were loaded and fractionated by SDS-PAGE under reducing conditions with β-mercaptoethanol. The separated proteins were transferred to polyvinylidene fluoride (PVDF) membranes, which were blocked (blocking conditions in Table 1), incubated with the primary antibody, washed, incubated with the secondary antibody (antibody information in Table 1), again washed, and then incubated with the WesternBright Chemiluminescence Substrate (Advansta). Protein bands were detected with the ChemiDoc MP Imaging system (Biorad). Protein band intensities were analyzed using the Image Lab software (Biorad), and normalized to housekeeper proteins as loading controls. β-actin (1:2000, ab8229, Abcam) or vinculin (1:1000, sc-73614, Santa Cruz Biotechnology) served as loading controls. To minimize heterogeneities due to variabilities between membranes, protein band intensities were additionally normalized to the mean protein band intensity of the CD group of the respective membrane and are thus shown as a percentage of the CD mean. The analyzed western blot membranes are shown in the supplement (Supplemental Figures 1–12).

**TABLE 1:** Western blot conditions.

Target/size	Loaded protein amount (μg)	Gel percentage (%)	Blocking conditions	Primary and secondary antibodies
Elastin (68 kDa)	30	12	Everyblot Blocking Buffer (Biorad) for 5 min	Anti-elastin (1:1000, ab-217356, Abcam)
				Anti-goat (1:10,000, sc-2020, Santa Cruz)
Fibrillin (312 kDa)	20	5	TBS for 30 s, drying	Anti-fibrillin (1:1000, ab-231094, Abcam)
				Anti-rabbit (1:10,000, 111-036-144, Dianova, Hamburg, Germany)
COL1A1 (70–90 kDa)	20	8	Everyblot Blocking Buffer (Biorad) for 5 min	Anti-COL1A1 (1:1000, ab-21286, Abcam)
				Anti-rabbit (1:10,000, 111-036-144, Dianova)
COL3A1 (110/140 kDa)	20	8	Everyblot Blocking Buffer (Biorad) for 5 min	Anti-COL3A1 (1:1000, ab-184993, Abcam)
				Anti-rabbit (1:10,000, 111-036-144, Dianova)

### Statistics

Data were analyzed using SigmaPlot software (Version 13, Systat Software GmbH). Data distribution was assessed for normality using the Kolmogorov–Smirnov test and for equality of variances using the Brown–Forsythe test. Two-way ANOVA was performed, followed by post-hoc Tukey test. *P* values <0.05 were considered statistically significant, and were indicated by ∗ = *P <* 0.05, ∗∗ = *P <* 0.01, and ∗∗∗ = *P <* 0.001. *P* values between 0.07 and 0.1 were considered to show a tendency to significance [46] and are shown if indicated in figure legends. Graphs were created using the GraphPad Prism software (Version 7, Dotmatics) and figures with Photoshop CS6 software (Adobe). Data are shown as individual values or as means ± SD as stated in the figure legends.

## Results

**FIGURE 2:**
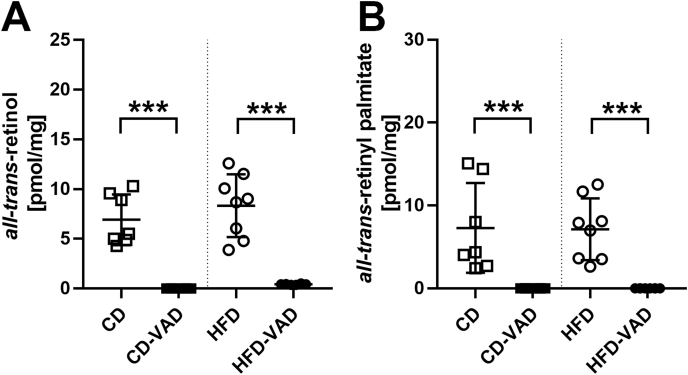
Vitamin A metabolites in the lung. (A) *all-trans-*retinol, (B) *all-trans-*retinyl palmitate. Data are presented as values of individual mice; group means and SDs are indicated. Statistics: 2-way ANOVA followed by post-hoc Tukey test; significant differences are indicated by ∗*P <* 0.05, ∗∗*P <* 0.01, and ∗∗∗*P <* 0.001. ANOVA, analysis of variance; CD, control diet; HFD, high-fat diet; VAD, vitamin A deficiency.

HFD-fed mice had higher body weights compared with CD-fed mice after 20 wk of dietary treatment, irrespective of VAD (see Figure 1E in [8]). Concentrations of *all-trans-*retinol and *all-trans-*retinyl palmitate were strongly reduced in lung tissue of CD-VAD and HFD-VAD groups (Figure 2), demonstrating an efficient depletion of pulmonary vitamin A reserves irrespective of the diet. The level of *all-trans-*retinoic acid was below the detection limit in all groups (not shown). Hepatic *all-trans-*retinol concentrations reflecting systemic retinol reserves [47] were emptied in both CD-VAD and HFD-VAD groups, similar to the lung (see Figure 1D in [8]). In contrast, adipose tissue *all-trans-*retinol amounts were depleted in CD-VAD, but not in HFD-VAD (Supplemental Figure 13).

### Lung structure and parenchyma composition

**FIGURE 3:**
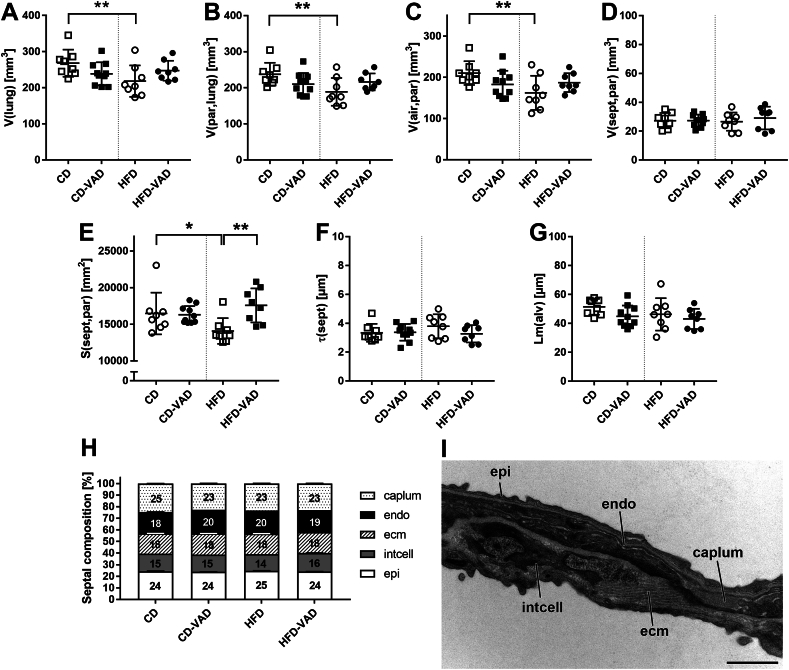
Lung structure. (A) lung volume, (B) parenchyma volume, (C) airspace volume, (D) septal volume, (E) alveolar surface area, (F) septal thickness, (G) mean linear intercept, (H) volume densities of septal compartments, (I) representative transmission electron microscopy image illustrating septal compartments, that is, epithelial cells (epi), interstitial cells (intcell), extracellular matrix (ecm), endothelial cells (endo), capillary lumen (caplum). Data are presented as values of individual mice; group means and SDs are indicated. Statistics: 2-way ANOVA followed by post-hoc Tukey test; significant differences are indicated by ∗*P <* 0.05, ∗∗*P <* 0.01, and ∗∗∗*P <* 0.001. Scale bar = 1 μm. ANOVA, analysis of variance; air, airspace; CD, control diet; HFD, high-fat diet; Lm, mean alveolar intercept; par, parenchyma; S, surface area; sept, septum; V, total volume; VAD, vitamin A deficiency; *τ*, thickness.

To detect diet- and VAD-related effects on the lung, the pulmonary structure was further evaluated. Results of all structural parameters are shown in the supplement (Supplemental Table 2). Left lung volumes and parenchymal volumes (parenchyma = lung compartment involved in gas exchange) were reduced in the HFD group compared with CD (Figure 3A, B). This decrease was due to a reduction in airspace, but not septal volume (Figure 3C, D), and was accompanied by a decreased septal surface area (Figure 3E). These HFD-related alterations were absent in the HFD-VAD and CD-VAD groups. The mean linear intercept as a measure for alveolar size and the septal thickness were similar in all groups (Figure 3F, G). Moreover, the ultrastructural composition of the septa, which consists of epithelial cells, ECM, interstitial cells (mainly fibroblasts), endothelial cells, and capillary lumen (Figure 3I), was neither altered by diet nor VAD (Figure 3H).

### ECM composition

**FIGURE 4:**
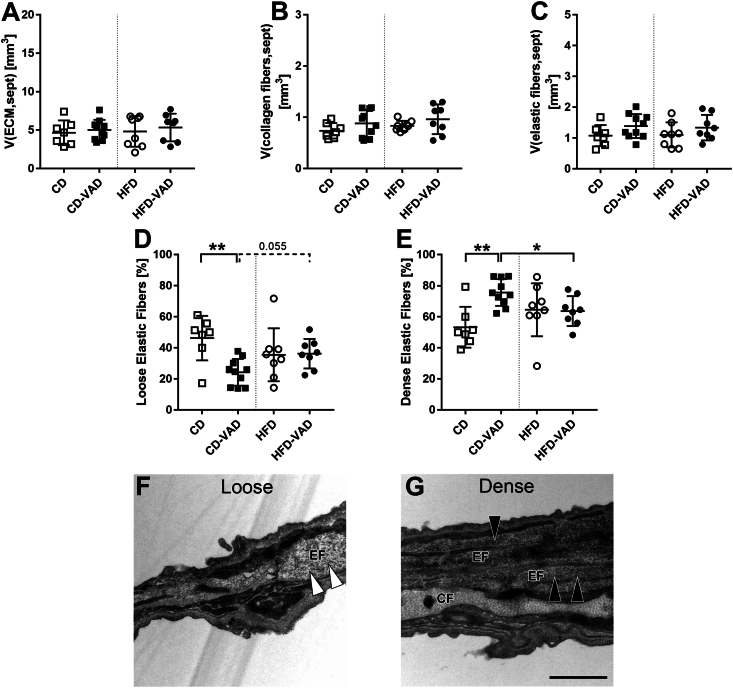
ECM composition. (A) ECM volume, (B) collagen fiber volume, (C) elastic fiber volume within the alveolar septa. (D–E) Scoring of elastic fiber appearance, (F–G) representative transmission electron microscopy images showing loose (white arrows) and dense (black arrows) structural composition of elastic fibers; F, CD; G, CD-VAD. Scale bar = 1 μm. Data are presented as values of individual mice; group means and SDs are indicated. Statistics: 2-way ANOVA followed by post-hoc Tukey test; significant differences are indicated by ∗*P <* 0.05, ∗∗*P <* 0.01, and ∗∗∗*P <* 0.001, tendencies are indicated by dashed lines and numerical *P* values. ANOVA, analysis of variance; CD, control diet; ECM, extracellular matrix; HFD, high-fat diet; sept, septum; VAD, vitamin A deficiency; V, total volume.

Volumes of the septal ECM and its fibrillar components, collagen and elastic fibers, were neither affected by diet nor VAD (Figure 4A–C). Elastic fibers consist of an elastin core and microfibrillar scaffold and can show a rather lose or dense appearance, likely depending on the abundance of microfibrils [48] (Figure 4F, G). Semiquantitative scoring revealed that densely packed elastic fibers were increased in CD-VAD compared with CD and HFD-VAD at the expense of loosely arranged elastic fibers (Figure 4E), indicating elastic fiber remodeling. These alterations in elastic fiber ultrastructure were not observed in the HFD-VAD group.

**FIGURE 5:**
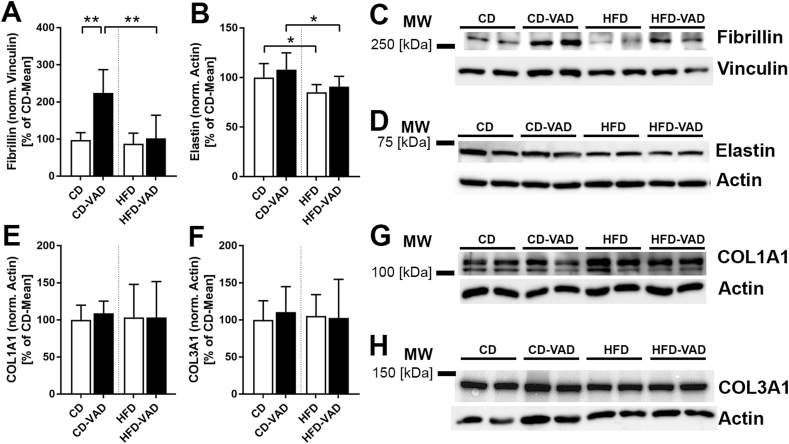
Protein expression of ECM components. (A, B, E, F) Quantification of protein expression; (C, D, G, H) representative western blot images, molecular marker bands are indicated. (A, B, E, F) Protein band intensities were normalized to respective housekeeping proteins and are shown as a percentage of the control group mean of the respective membrane. Data are presented as group means (CD, *n =* 6; CD-VAD, *n =* 4; HFD, *n =* 7; HFD-VAD, *n =* 6); SDs are indicated. Statistics: 2-way ANOVA followed by post-hoc Tukey test; significant differences are indicated by ∗*P <* 0.05, ∗∗*P <* 0.01, and ∗∗∗*P <* 0.001. ANOVA, analysis of variance; CD, control diet; ECM, extracellular matrix; HFD, high-fat diet; VAD, vitamin A deficiency.

These observations were in line with a significantly increased protein expression of fibrillin, which is the main component of microfibrils in the lungs [48], of CD-VAD compared with CD and HFD-VAD (Figure 5A, C). Besides, elastin expression was reduced in HFD-fed mice irrespective of VAD (Figure 5B, D). In contrast, pulmonary protein expression of collagen I and III was similar between the groups (Figure 5E–H).

### Septal lipid content and AE2 cells

**FIGURE 6:**
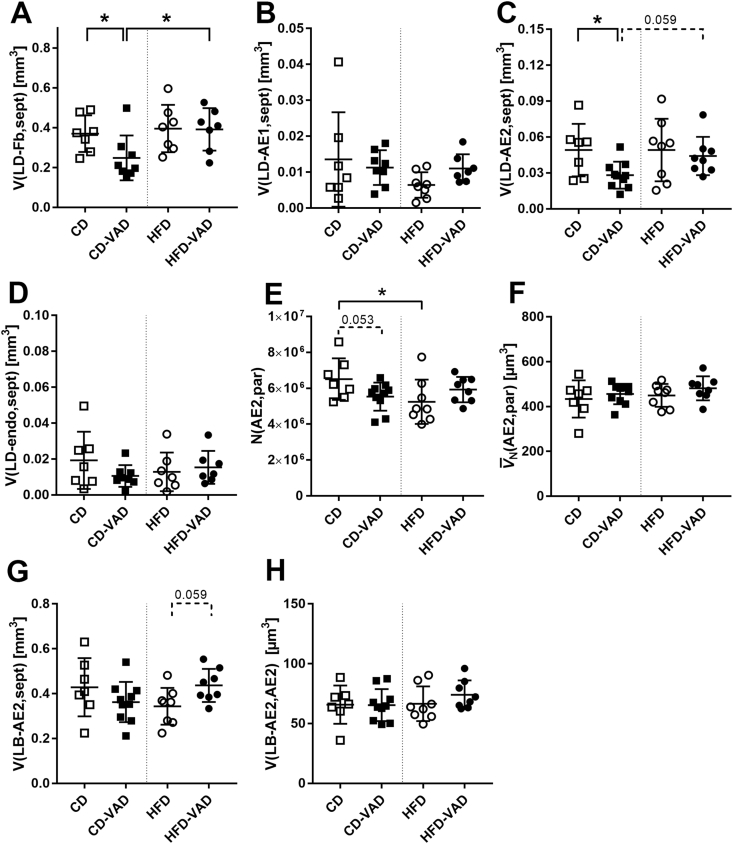
Septal lipid content and AE2 cell alterations. Total volumes of lipid droplets in (A) fibroblasts, (B) AE1 cells, (C) AE2 cells, (D) endothelial cells related to the alveolar septa. (E) Total number of parenchymal AE2 cells, (F) number-weighted mean volume per AE2 cell. Total volumes of (G) lamellar bodies in AE2 cells related to the alveolar septa and (H) related to the number-weighted mean volume of AE2 cells. Data are presented as values of individual mice; group means and SDs are indicated. Statistics: 2-way ANOVA followed by post-hoc Tukey test; significant differences are indicated by ∗*P <* 0.05, ∗∗*P <* 0.01, and ∗∗∗*P <* 0.001, tendencies are indicated by dashed lines and numerical *P* values. AE1, alveolar epithelial type 1 cell; AE2, alveolar epithelial type 2 cell; ANOVA, analysis of variance; CD, control diet; endo, endothelial cell; Fb, fibroblast; HFD, high-fat diet; LB, lamellar body; LD, lipid droplet; N, number; par, parenchyma; sept, septum; V, total volume; VAD, vitamin A deficiency.

Lipid droplet volumes in septal fibroblasts decreased in CD-VAD compared with CD and HFD-VAD (Figure 6A). Regarding septal epithelial cells, CD-VAD induced a reduction in lipid volumes only in AE2, but not in AE1 cells (Figure 6B, C). The lipid content in endothelial cells was not affected by diet or VAD (Figure 6D).

A further evaluation of AE2 cells revealed that CD-VAD resulted in a strong tendency to reduced cell numbers, whereas the HFD significantly decreased the number of AE2 cells (Figure 6E). The AE2 cell mean volume was comparable between the groups (Figure 6F). Although the total volume of lamellar bodies per lung showed a slight tendency to be reduced in HFD (Figure 6G), the lamellar body volume per AE2 cell was not changed by any experimental condition (Figure 6H).

## Discussion

The present study investigated the potential cumulative effects of VAD and HFD on the pulmonary gas-exchange region. The utilized mouse model combined an impaired vitamin A storage due to an LRAT-knockout with a vitamin A deficient control or fat-enriched diet, and resulted in an efficient depletion of vitamin A in the lungs of lean or obese mice. Both experimental conditions, that is, obesity and VAD, induced individual changes in the lung parenchyma. Obesity led to reduced lung and parenchymal volumes, which was due to decreased airspace, but not septal volumes. Moreover, AE2 cell numbers were diminished in HFD-fed mice. VAD in lean mice resulted in a higher percentage of septal elastic fibers that appeared ultrastructurally densely packed. This was associated with increased fibrillin protein expression, indicating elastic fiber remodeling. Moreover, lipid droplet volumes were decreased in septal fibroblasts and AE2 cells, and AE2 cell numbers showed a strong tendency to be decreased in CD-VAD. These obesity- or VAD-related alterations were attenuated or even absent in obese VAD mice.

### HFD-related effects

As previously described by others, obesity resulted in reduced parenchymal and airspace volumes (Figure 3B, C), likely induced by accumulation of adipose tissue in the mediastinum as well as thoracic and abdominal cavities [49,50]. Moreover, AE2 cell numbers were decreased (Figure 6E), indicating an apoptotic stimulus of the HFD. Although these changes were absent in HFD-VAD, pulmonary elastin expression was decreased in both HFD and HFD-VAD groups (Figure 5B, D), albeit without obvious impact on the ultrastructural appearance or the absolute volume of septal elastic fibers (Figure 4C–E).

### ECM-related effects of VAD

The pulmonary ECM consists mainly of elastic fibers, collagen fibers, and proteoglycans, located in the interstitial space of the alveolar septa [51]. Subsequent post-translational modifications are essential for the structure and biomechanics of the fibrillar components [52]. Elastic fibers are the largest structures of the ECM and are critical in providing elasticity, recoil, and structural integrity against mechanical stress [53,54]. They consist of 2 components: an elastin core and a scaffold of microfibrils, of which fibrillin-1 is the major structural element [48]. In the present study, VAD resulted in increased fibrillin expression in CD-fed mice (Figure 5 A, C), accompanied by a dense ultrastructural appearance of elastic fibers (Figure 4 D), directly pointing to a higher abundance of electron-dense microfibrils in the elastic fibers. Functionally, microfibril-rich elastic fibers are associated with altered elastic recoil properties affecting lung ventilation, that is, a higher stiffness in aged lungs [55]. The assembly of elastic fibers is critically dependent on the presence of fibrillin, and the disruption of microfibril assembly has profound effects on elastogenesis due to impaired tissue integrity [53,56]. Mice lacking fibrillin-1 exhibit a loss of cell attachments, accompanied by thinning and fragmentation of elastic fibers within the aorta, indicating elastolysis and remodeling of elastic fibers [57]. A previous report has shown that high sucrose intake in mice is associated with a mismatch between high elastin and comparatively low fibrillin-1 protein expression, as well as a reduced abundance of microfibrils in elastic fibers. This in turn leads to a reduced lung elasticity, like a worn-out balloon [45]. Because the CD-VAD mice tend to have more microfibrils, this could indicate a higher stiffness of the lungs.

In patients with emphysema, pulmonary fibrillin-1 expression is increased and positively correlated with distal airspace enlargement as well as the degree of parenchymal destruction, suggesting that elevated fibrillin-1 expression is associated with the early onset of lung emphysema [58]. Also, VAD might be linked to emphysematous changes in the lung, because vitamin A-deficient nutrition for 6 wk induces enlarged distal airspaces and partial destruction of the alveolar septa in rats [18]. Although we could not detect any signs of septal degradation in the CD-VAD group (Figure 3D), we cannot exclude the possibility that pulmonary emphysema, possibly triggered by the high fibrillin-1 abundance, might have occurred at a later time point.

The protein expression levels of collagen I and III, the most abundant collagen types in the lung parenchyma [59], were comparable between the groups in the present study. This is in contrast to previously described effects of VAD on collagen fibers. In newborn rats, vitamin A-deficient diet for 9 wk leads to increased pulmonary protein and mRNA expression of collagen I measured by western blot and qRT-PCR, as well as a thickening of the alveolar basement membrane due to ectopic collagen I deposition [20]. On the other hand, in mice with diet-induced VAD for 7–10 wk, the amount of septal collagen fibers was significantly decreased [23]. This discrepancy might be due to the used animal models (almost complete depletion of vitamin A reserves due to LRAT-knockout in this study compared with vitamin A-deficient diets; feeding duration 20 wk in this study compared with ∼10 wk) and detection methods.

### Lipid- and AE2 cell-related alterations of VAD

Vitamin A is stored as retinyl esters, including *all-trans-*retinyl palmitate and—stearate, incorporated into lipid droplets of various cell types such as hepatic stellate cells or adipocytes [13,60]. Within the lung, primarily neutral lipids within fibroblasts, alveolar epithelial cells (AE1 and AE2 cells), and endothelial cells serve as storage for vitamin A [3]. In the current study, CD-VAD decreased lipid droplet volume in the cell types mainly implicated in alveolar lipid metabolism, namely fibroblasts and AE2 cells, but not in AE1 or endothelial cells (Figure 6 A, C). Fibroblasts are the main producers of the ECM and, at least in mice, involved in the storage and provision of lipids [61,62], whereas AE2 cells maintain an active lipid metabolism for surfactant synthesis [63]. In vitro data suggest that fibroblasts shuttle triglycerides to AE2 cells, which in turn use these for production of surfactant-associated lipid species [64], indicating a close cooperation of these cell types. The lower lipid volumes under VAD are in line with a study reporting decreased serum and hepatic lipid concentrations after diet-induced VAD for 12 wk in rats [65]. Moreover, in vitro experiments in hepatic stellate cells show that LRAT and retinyl esters independently drive lipid droplet formation, and a reduced LRAT expression results in the absence of retinyl esters containing lipid droplets [[66], [67], [68]]. Our data indicate that similar regulatory mechanisms occur in alveolar fibroblasts and AE2 cells, demonstrating a direct impact of the systemic vitamin A metabolism on the lung. In the same line of evidence, Shamarakov et al. [3] showed that depletion of alveolar retinyl ester stores due to LRAT-knockout significantly increases the severity of LPS-induced acute lung injury, including reduced survival and aggravated infiltration of inflammatory cells. These results point to a clinical relevance of VAD in the context of specific lung diseases.

### VAD and obesity

The alveolar changes induced by obesity or VAD were attenuated or even absent when both conditions occurred together; thus, the initial hypothesis was not supported by the results of this study. One possible explanation could be that the decrease in lipid volumes in fibroblasts was directly linked to the elastic fiber remodeling under CD-VAD, and that the obese status in HFD-VAD somehow prevents this lipid reduction by higher systemic lipid reserves. Moreover, retinol levels were preserved under HFD-VAD in white adipose tissue depots (Supplemental Figure 13). It is unclear to what extent these retinol residues could have influenced the VAD-related pulmonary effects. It is known that in adipose tissue retinoids are utilized preferentially in an autocrine manner for local metabolic processes, including adipocyte differentiation and regulation of insulin sensitivity [13,69,70]. Moreover, systemic (reflected by hepatic retinol concentrations, Figure 1D in [8]) and local (pulmonary, Figure 2) retinoids were efficiently depleted under VAD conditions in both CD- and HFD-fed mice. Nevertheless, we cannot rule out that retinoid residues located in adipose tissue might have been transferred to the lung, thereby affecting the assessed pulmonary parameters.

In addition to that, other factors may also play a role. Several mouse models indicate a link between vitamin A metabolism and the development of obesity, as the genetic knockout of enzymes of the vitamin A metabolism had adipogenic effects [9,[71], [72], [73], [74]]. One phenomenon currently discussed is the so-called “obesity paradox”, saying that despite the various adverse effects of obesity, there are also protective effects under chronic conditions. Several reasons for this are being discussed, including higher fat and muscle reserves or an adaptation to a low-grade inflammation leading to attenuated inflammatory responses to acute inflammation [75,76]. It is currently unclear whether similar effects play a role for VAD.

In conclusion, pulmonary depletion of vitamin A reserves resulted in fibrillin overexpression and elastic fiber remodeling, as well as lipid volume decreases in fibroblasts and AE2 cells within the alveolar niche. Diet-induced obesity reduced lung volumes and AE2 cell numbers. Unexpectedly, these VAD- and obesity-related effects were attenuated or even abrogated when the combination of the conditions was present. Future studies are required to investigate the underlying mechanisms for these mutually mitigating effects of VAD and obesity.

## Author contributions

The authors’ responsibilities were as follows – L-MH, NF, CR, JS: conceived and designed the experiments; MB, LN, HB, CR, JS: performed the experiments; L-MH, TM, NM, CM: analyzed the data; L-MH, JS: wrote the original manuscript; and all authors: read and approved the final manuscript for submission.

## Data availability

The data supporting this article have been included as part of the Supplementary Data.

## Funding

This research was supported by German Research Foundation Research Grant RI 2417/4-1 (to CR), and a Kaltenbach fellowship of the German Heart Foundation (Deutsche Herzstiftung) (to LN).

## Conflict of interest

CR reports financial support was provided by German Research Foundation. LN reports financial support was provided by German Heart Foundation. Other authors report no conflict of interest.
