# Supplementary material for: Dietary Fat Intake Attenuates Vitamin A Deficiency-Associated Elastic Fiber Remodeling and Lipid Reduction in the Alveolar Niche in Mice
Source: J Nutr. 2025 Jul 17;155(10):3455–65. doi: 10.1016/j.tjnut.2025.07.010 (PMC12799404; doi:10.1016/j.tjnut.2025.07.010)
Supplement: Multimedia component 1 [file mmc1.docx]

Supplementary Data

Dietary fat intake attenuates vitamin A deficiency-associated elastic fiber remodeling and lipid reduction in the alveolar niche in mice

Lisa-Marie Hoy^1^, Tabea Meier^1^, Natascha Mierswa^1^, Melanie Bornemann^1^, Lea Naasner^2^, Heike Bähre^3^, Natali Froese^2^, Christian Riehle^2^, Christian Mühlfeld^1,4^, Julia Schipke^1,4^

^1^ Hannover Medical School, Institute of Functional and Applied Anatomy, Carl-Neuberg-Str. 1, 30625 Hannover, Germany

^2^ Hannover Medical School, Department of Cardiology and Angiology, Carl-Neuberg-Str. 1, 30625 Hannover, Germany

^3^ Research Core Unit Metabolomics, Institute of Pharmacology, Hannover Medical School, Hannover, Germany

^4^ Biomedical Research in Endstage and Obstructive Lung Disease Hannover (BREATH), Member of the German Center for Lung Research (DZL), 30625 Hannover, Germany

**Corresponding author**

PD Dr. Julia Schipke

Institute of Functional and Applied Anatomy, Hannover Medical School, Carl-Neuberg-Str. 1,

30625 Hannover, Germany

Tel.: +49 511 532 2997

E-Mail: schipke.julia@mh-hannover.de

**Table S1. Composition of the animal diets.**

| **Ingredient** | **Unit** | **CD** | **HFD** | **CD-VAD** | **HFD-VAD** |
| --- | --- | --- | --- | --- | --- |
| **Carbohydrate (total)** | **[% by calories]** | **67.9** | **23.2** | **67.8** | **22.5** |
| Monosaccharides | [% by calories] | - | 7.9 | - | 8.3 |
| Disaccharides | [% by calories] | 13.0 | 3.4 | 11.4 | 2.4 |
| Polysaccharides | [% by calories] | 54.9 | 11.8 | 56.4 | 11.8 |
| **Protein (total)** | **[% by calories]** | **20.1** | **16.5** | **20.1** | **16.6** |
| Alanine | [g/kg] | 2.53 | 11.68 | 2.53 | 11.76 |
| Arginine | [g/kg] | 9.83 | 13.33 | 9.83 | 13.43 |
| Aspartic acid | [g/kg] | 3.58 | 12.26 | 3.59 | 12.45 |
| Cystine | [g/kg] | 3.20 | 1.41 | 3.20 | 1.43 |
| Glutamic acid | [g/kg] | 23.67 | 29.68 | 23.69 | 30.18 |
| Glycine | [g/kg] | 3.14 | 24.10 | 3.14 | 24.25 |
| Histidine | [g/kg] | 5.28 | 3.42 | 5.28 | 3.48 |
| Isoleucine | [g/kg] | 7.22 | 7.13 | 7.23 | 7.30 |
| Leucine | [g/kg] | 14.76 | 7.48 | 14.77 | 7.52 |
| Lysine | [g/kg] | 17.40 | 11.75 | 17.40 | 11.91 |
| Methionine | [g/kg] | 7.22 | 4.32 | 10.69 | 4.41 |
| Phenylalanine | [g/kg] | 7.17 | 7.04 | 7.18 | 7.16 |
| Proline | [g/kg] | 12.76 | 20.46 | 12.77 | 20.67 |
| Serine | [g/kg] | 5.27 | 8.12 | 5.27 | 8.25 |
| Threonine | [g/kg] | 7.15 | 6.16 | 7.16 | 6.27 |
| Tryptophan | [g/kg] | 1.98 | 1.25 | 1.98 | 1.29 |
| Tyrosine | [g/kg] | 9.29 | 5.12 | 9.29 | 5.23 |
| Valine | [g/kg] | 3.30 | 7.49 | 3.30 | 7.64 |
| **Fat (total)** | **[% by calories]** | **13.0** | **60.1** | **13.0** | **60.8** |
| Saturated fatty acids | [% by calories] | 1.2 | 21.8 | 1.2 | 22.0 |
| Monounsaturated fatty acids | [% by calories] | 3.5 | 21.3 | 3.5 | 21.4 |
| Polyunsaturated fatty acids | [% by calories] | 7.3 | 4.3 | 7.3 | 4.3 |
| C4, Butyric | [g/kg] | - | 4.19 | - | 4.36 |
| C7, Caproic | [g/kg] | - | 3.68 | - | 3.83 |
| C8, Caprylic | [g/kg] | - | 1.27 | - | 1.32 |
| C10, Capric | [g/kg] | - | 2.62 | - | 2.72 |
| C12, Lauric | [g/kg] | 0.05 | 3.13 | 0.05 | 3.25 |
| C14, Myristic | [g/kg] | 0.10 | 13.90 | 0.10 | 14.34 |
| C16, Palmitic | [g/kg] | 2.50 | 56.56 | 2.50 | 56.67 |
| C17, Margaric | [g/kg] | 0.10 | 0.43 | 0.10 | 0.43 |
| C18, Stearic | [g/kg] | 1.35 | 37.17 | 1.35 | 37.49 |
| C20, Arachidic | [g/kg] | 0.05 | 3.79 | 0.05 | 3.79 |
| C22, Behenic | [g/kg] | 0.15 | - | 0.15 | - |
| C24, Lignoceric | [g/kg] | 0.25 | - | 0.25 | - |
| C16:1, Palmitoleic | [g/kg] | 0.15 | 8.90 | 0.15 | 8.99 |
| C18:1, Oleic | [g/kg] | 13.50 | 111.56 | 13.50 | 112.45 |
| C-20:1, Eicosaenoic | [g/kg] | 0.15 | 1.71 | 0.15 | 1.71 |
| C22:1, Erucic | [g/kg] | 0.05 | 1.75 | 0.05 | 1.82 |
| C18:2, Linoleic | [g/kg] | 28.50 | 22.12 | 28.50 | 22.17 |
| C18:3, Linolenic | [g/kg] | 0.15 | 3.13 | 0.15 | 3.16 |
| **Supplements** | | | | | |
| Cholesterol | [mg/kg] | - | 211.85 | - | 211.85 |
| Chondroitin | [g/kg] | - | 31.20 | - | 31.20 |
| Glucosamine | [g/kg] | - | 66.00 | - | 66.00 |
| Inositol | [mg/kg] | 111.00 | 101.93 | 111.00 | 101.93 |
| **Vitamins** | | | | | |
| Biotin | [mg/kg] | 0.20 | 0.20 | 0.20 | 0.20 |
| Choline chloride | [g/kg] | 1.01 | 1.00 | 1.01 | 1.00 |
| Folic acid | [mg/kg] | 10.00 | 10.02 | 10.01 | 10.02 |
| Nicotinic acid | [mg/kg] | 50.17 | 50.03 | 50.17 | 50.03 |
| Pantothenic acid | [mg/kg] | 50.11 | 50.02 | 50.11 | 50.02 |
| Vitamin A | [I.E./kg] | 15.000 | 15.000 | - | - |
| Vitamin B1 | [mg/kg] | 20.04 | 20.01 | 20.04 | 20.01 |
| Vitamin B2 | [mg/kg] | 20.32 | 20.06 | 20.32 | 20.06 |
| Vitamin B6 | [mg/kg] | 15.23 | 15.21 | 15.03 | 15.01 |
| Vitamin B12 | [mg/kg] | 0.04 | 0.03 | 0.04 | 0.03 |
| Vitamin C | [mg/kg] | 20.00 | 20.00 | 21.00 | 21.00 |
| Vitamin D3 | [I.E./kg] | 500.00 | 500.00 | 500.00 | 500.00 |
| Vitamin E | [mg/kg] | 180.40 | 150.07 | 180.40 | 150.07 |
| Vitamin K3 | [mg/kg] | 10.20 | 10.00 | 10.20 | 10.00 |
| **Minerals** | | | | | |
| Aluminum | [mg/kg] | 3.71 | 2.37 | 3.74 | 2.45 |
| Calcium | [g/kg] | 9.31 | 7.21 | 9.54 | 7.28 |
| Chlorine | [g/kg] | 3.63 | 3.93 | 3.63 | 4.00 |
| Cobalt | [mg/kg] | 0.15 | 0.09 | 0.15 | 0.09 |
| Copper | [mg/kg] | 5.75 | 3.32 | 5.65 | 3.32 |
| Fluorine | [mg/kg] | 4.17 | 3.03 | 4.17 | 3.03 |
| Iodine | [mg/kg] | 0.51 | 0.27 | 0.45 | 0.27 |
| Iron | [mg/kg] | 178.58 | 104.43 | 178.61 | 104.42 |
| Magnesium | [g/kg] | 0.68 | 0.60 | 0.67 | 0.60 |
| Manganese | [mg/kg] | 100.89 | 58.94 | 100.89 | 58.95 |
| Molybdenum | [mg/kg] | 0.20 | 0.18 | 0.20 | 0.18 |
| Phosphorus (digested) | [g/kg] | 7.20 | 4.45 | 7.21 | 4.49 |
| Phosphorus (total) | [g/kg] | 7.52 | 5.35 | 7.53 | 5.41 |
| Potassium | [g/kg] | 7.09 | 6.58 | 7.17 | 6.67 |
| Selenium | [mg/kg] | 0.33 | 0.17 | 0.33 | 0.17 |
| Sodium | [g/kg] | 2.49 | 1.67 | 2.50 | 1.67 |
| Sulfur | [g/kg] | 2.79 | 1.42 | 2.79 | 0.68 |
| Zinc | [mg/kg] | 29.30 | 15.24 | 29.31 | 15.24 |

**Table S2. Results of structural analysis of the lung by design-based stereology.**

| **Parameter** | **CD** | **CD-VAD** | **HFD** | **HFD-VAD** |
| --- | --- | --- | --- | --- |
| V(lung) [mm^3^] | 268.00  *±37.40* | 238.50  *±31.84*  vs. CD p=0.089 | 218.38  *±43.41* vs. CD p=0.009 | 247.38  *±27.50* |
| V_V_(par/lung) | 0.89  *±0.01* | 0.88 *±0.03*  vs. CD p=0.077 | 0.87  *±0.03* vs. CD p=0.057 | 0.87  *±0.01*  vs. HFD p=0.095 |
| V(par,lung) [mm^3^] | 237.56  *±31.94* | 210.12  *±31.06*  vs. CD p=0.077 | 188.95  *±38.36*  vs. CD p=0.005 | 216.21 *±23.50*  vs. HFD p=0.095 |
| **Parenchymal composition** | | | | |
| V_V_(air/par) | 0.86 *±0.02* | 0.87  *±0.03* | 0.85  *±0.06*  vs. CD p=0.083 | 0.87 *±0.04* |
| V_V_(sept/par) | 0.12  *±0.02* | 0.13  *±0.03* | 0.15 *±0.06*  vs. CD p=0.083 | 0.14  *±0.04* |
| V(air,par) [mm^3^] | 201.5  *±15.8* | 182.8  *±33.1*  vs. CD p=0.083 | 162.3  *±41.5*  vs. CD p=0.006 | 187.1  *±22.9* |
| V(sept,par) [mm^3^] | 27.30  *±5.30* | 27.35  *±4.16* | 26.66 *±6.27* | 29.12 *±7.94* |
| S_V_(sept/par) [mm^-1^] | 69.40  *±7.26* | 78.79 *±10.26*  vs. CD p=0.069 | 76.44 *±13.56* | 81.59 *±9.87* |
| S(sept,par) [mm^2^] | 15537 ±*1119* | 16307  ±*1179* | 13518 ±*778*  vs. CD p=0.028 | 17574 ±*2326*  vs. HFD p=0.002 |
| 𝜏(sept) [µm] | 3.15  *±0.33* | 3.37  *±0.58* | 3.79  *±0.83* | 3.27  *±0.60* |
| Lm(alv) | 51.5  *±5.4* | 44.9  *±7.5*  vs.CD p=0.091 | 46.3  *±11.2* | 43.1  *±7.0* |
| **Septal composition** | | | | |
| V_V_(AE1/sept) | 0.153  *±0.019* | 0.162  *±0.020* | 0.160  *±0.016* | 0.143  *±0.016*  vs. CD-VAD p=0.026  vs. HFD p=0.053 |
| V_V_(AE2/sept) | 0.092  *±0.025* | 0.076 *±0.023* | 0.087  *±0.030* | 0.097 *±0.022*  vs. CD-VAD p=0.078 |
| V_V_(epi/sept) | 0.244  *±0.034* | 0.238  *±0.031* | 0.247  *±0.044* | 0.240  *±0.028* |
| V_V_(intcell/sept) | 0.151  *±0.025* | 0.149  *±0.020* | 0.137  *±0.024* | 0.161  *±0.013* |
| V_V_(ECM/sept) | 0.175  *±0.041* | 0.182  *±0.027* | 0.176  *±0.049* | 0.180  *±0.017* |
| V_V_(endo/sept) | 0.184  *±0.020* | 0.203  *±0.018*  vs. CD-VAD p=0.068 | 0.199  *±0.026* | 0.189  *±0.017* |
| V_V_(caplum/sept) | 0.25 *±0.02* | 0.22 *±0.04* | 0.23 *±0.03* | 0.23 *±0.03* |
| V(AE1,sept) [mm^3^] | 4.03 *±1.06* | 4.43 *±0.85* | 3.92 *±0.54* | 4.17 *±1.30* |
| V(AE2,sept) [mm^3^] | 2.38  *±0.67* | 2.10  *±0.79* | 1.97 *±0.31* | 2.78 *±0.84*  vs. CD-VAD p=0.083 |
| V(epi,sept) [mm^3^] | 6.42 *±1.49* | 6.53  *±1.44* | 5.89 *±0.39* | 6.94 *±1.98* |
| V(intcell,sept) [mm^3^] | 3.93 *±0.80* | 4.08  *±0.78* | 3.86  *±1.16* | 4.68  *±1.54* |
| V(ECM,sept) [mm^3^] | 4.65  *±1.61* | 5.02  *±1.32* | 4.83  *±1.99* | 5.34  *±1.80* |
| V(endo,sept) [mm^3^] | 4.74 *±0.51* | 5.53  *±0.84* | 5.37  *±1.67* | 5.50  *±1.55* |
| V(caplum,sept) [mm^3^] | 6.44 *±1.35* | 6.19  *±1.16* | 6.10  *±0.94* | 6.66  *±1.97* |
| **ECM composition** | | | | |
| V_V_(elast/sept) | 0.0103  *±0.0129* | 0.0110  *±0.0123* | 0.0410  *±0.0093* | 0.0463  *±0.0077* |
| V_V_(coll/sept) | 0.0048  *±0.0059* | 0.0064  *±0.0072* | 0.0324  *±0.0054* | 0.0332  *±0.0055* |
| V(elast,sept) [mm^3^] | 0.250  *±0.318* | 0.330 *±0.370* | 1.103 *±0.379* | 1.338 *±0.389* |
| V(coll,sept) [mm^3^] | 0.123  *±0.140* | 0.228 *±0.249* | 0.835 *±0.091* | 0.961 *±0.271* |
| elast/coll-ratio | 0.411  *±0.476* | 0.365  *±0.511* | 1.306  *±0.371* | 1.423  *±0.277* |
| **Septal lipids** | | | | |
| V_V_(LD-Fb/sept) | 0.0035  *±0.0037* | 0.0083  *±0.0032*  vs. CD p=<0.001 | 0.0140  *±0.0019* | 0.0137  *±0.0036*  vs. CD-VAD p=<0.001 |
| V_V_(LD-AE1/sept) | 0.0004  *±0.0005* | 0.0004  *±0.0002* | 0.0006  *±0.0001*  vs. CD p=0.039 | 0.0005  *±0.0001* |
| V_V_(LD-endo/sept) | 0.0004  *±0.0005* | 0.0005  *±0.0003* | 0.0004  *±0.0003* | 0.0005  *±0.0003* |
| V(LD-Fb,sept) [mm^3^] | 0.3703  *±0.0851* | 0.2531  *±0.0950*  vs. CD p=0.030 | 0.3774  *±0.1136* | 0.3852  *±0.0938*  vs. CD-VAD p=0.002 |
| V(LD-AE1,sept) [mm^3^] | 0.0095 *±0.0122* | 0.0111 *±0.0041* | 0.0127  *±0.0033* | 0.0118  *±0.0040* |
| V(LD-endo,sept) [mm^3^] | 0.0121  *±0.0147* | 0.0130  *±0.0086* | 0.0114  *±0.0101* | 0.0150  *±0.0080* |
| **AE2 cells** | | | | |
| N_V_(AE2/par) [/µm3] | 0.0000269  *±0.0000035* | 0.0000266  *±0.0000035* | 0.0000278 *±0.0000027* | 0.0000276 *±0.0000037* |
| N(AE2/par) | 6511575  *±1073095* | 5536106 *±735313*  vs. CD-VAD p=0.053 | 5248756 *±1154884*  vs. CD p=0.019 | 5928931 *±665898* |
| 𝑉̅_N_(AE2/par) [μm^3^] | 433.83 *±82.877* | 456.15 *±50.345* | 449.75 *±53.446* | 481.34 *±40.493* |
| V_V_(LD-AE2/AE2) | 0.019  *±0.006* | 0.010  *±0.002*  vs. CD p=0.020 | 0.020  *±0.007* | 0.015  *±0.005*  vs. CD-VAD p=0.089 |
| V_V_(LB-AE2/AE2) | 0.152  *±0.015* | 0.142  *±0.017* | 0.142  *±0.022* | 0.151  *±0.013* |
| V(LD-AE2,sept) [mm^3^] | 0.049 *±0.020* | 0.023  *±0.008*  vs. CD p=0.034 | 0.050  *±0.024* | 0.044  *±0.016*  vs. CD-VAD p=0.089 |
| V(LB-AE2, AE2) [µm^3^] | 65.74  *±14.81* | 63.64  *±11.92* | 62.96  *±14.44* | 74.88  *±11.19* |
| V(LB-AE2,sept) [mm^3^] | 0.434  *±0.120* | 0.333  *±0.064* | 0.331  *±0.079*  vs. CD p=0.094 | 0.431  *±0.071*  vs. HFD p=0.059 |

Data are presented as group means *±* SDs. Statistics: two-way ANOVA followed by post-hoc Tukey test; significant differences and tendencies are indicated as numerical p-values. CD, control diet; HFD, high-fat diet; VAD, vitamin A deficiency; V, total volume; V_V_, volume density; S_V_, surface density; S, surface area; 𝜏, thickness; Lm, mean alveolar intercept; N_V_, numerical density; N, number; 𝑉̅_N_ = number-weighted mean volume; sept, septum; air, airspace; alvair, alveolar airspace; ductair, ductal airspace; AE1, alveolar epithelial type 1 cell; AE2, alveolar epithelial type 2 cell; epi, epithelial cell; intcell, interstitial cell; ECM, extracellular matrix; endo, endothelial cell; caplum, capillary lumen; coll, collagen fiber; elast, elastic fiber; Fb, Fibroblast LD, lipid droplet; LB, lamellar body.


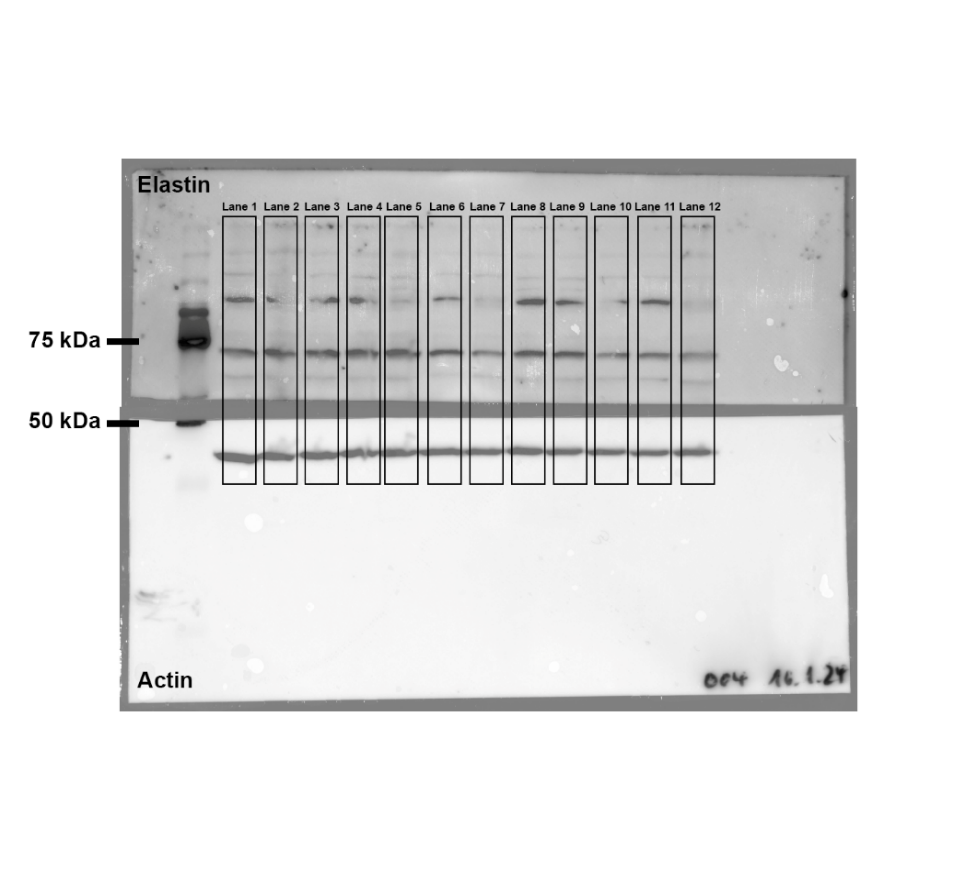


**Figure S1.** Original PVDF membrane for quantification of elastin expression. Upper panel: Elastin, lower panel: β-actin. Lane 1-3: HFD-VAD; Lane 8,9: CD; Lane 10-11: HFD; Lane 4-7, 12: other samples. Membrane was cut at 50 kDa for simultaneous development of target and housekeeper. Chemiluminescence and colorimetry (for marker bands) were merged with the Chemidoc MP imaging system.


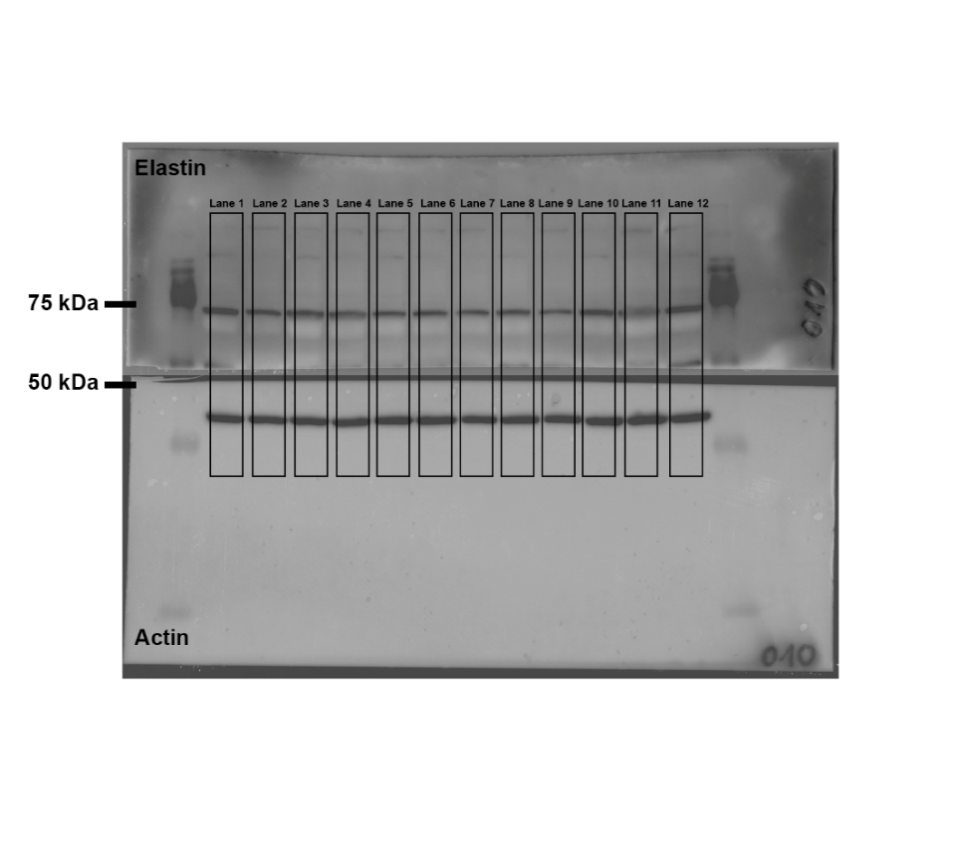


**Figure S2.** Original PVDF membrane for quantification of elastin expression. Upper panel: Elastin, lower panel: β-actin. Lane 1, 2: CD; Lane 3, 4: CD-VAD; Lane 5, 6: HFD; Lane 7, 8: HFD-VAD; Lane 9-12: other samples. Membrane was cut at 50 kDa for simultaneous development of target and housekeeper. Chemiluminescence and colorimetry (for marker bands) were merged with the Chemidoc MP imaging system.


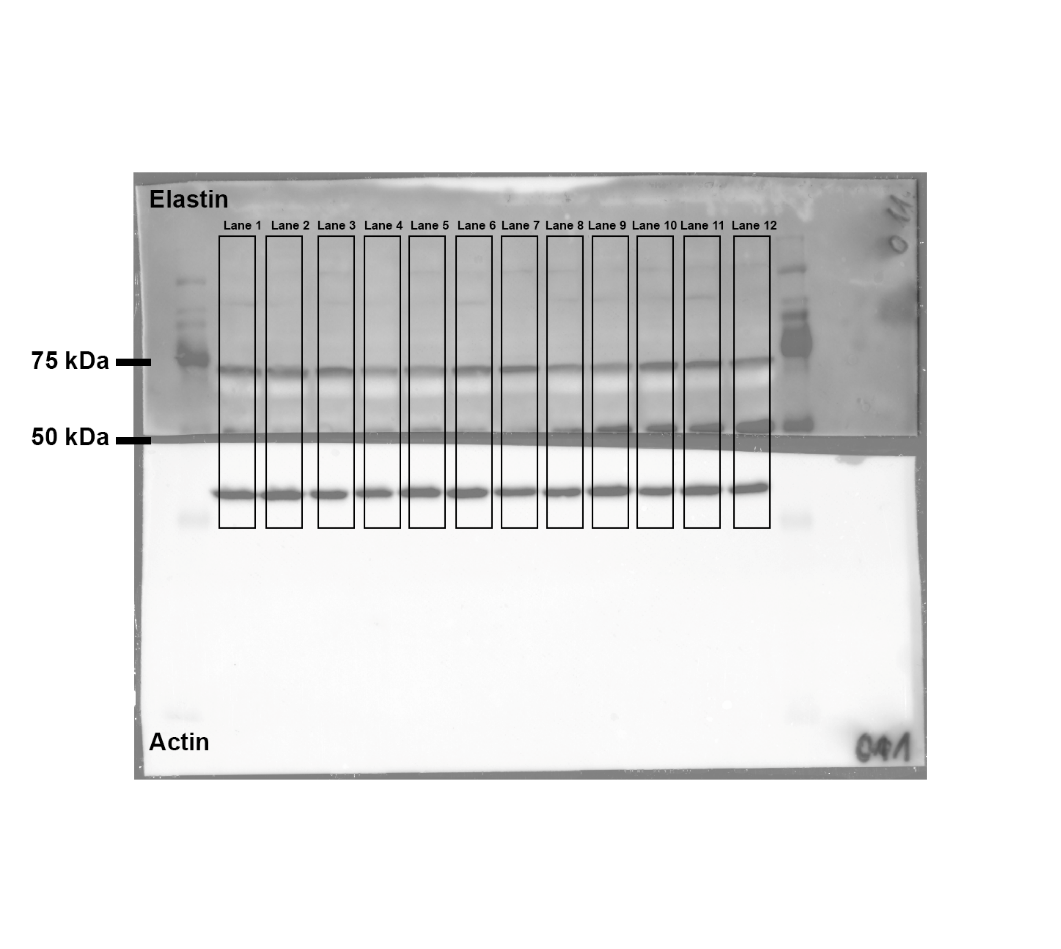


**Figure S3.** Original PVDF membrane for quantification of elastin expression. Upper panel: Elastin, lower panel: β-actin. Lane Lane 1, 2: CD; Lane 3, 4: CD-VAD; Lane 5, 6: HFD;Lane 7, 8: HFD-VAD; Lane 9-12: other samples. Membrane was cut at 50 kDa for simultaneous development of target and housekeeper. Chemiluminescence and colorimetry (for marker bands) were merged with the Chemidoc MP imaging system.


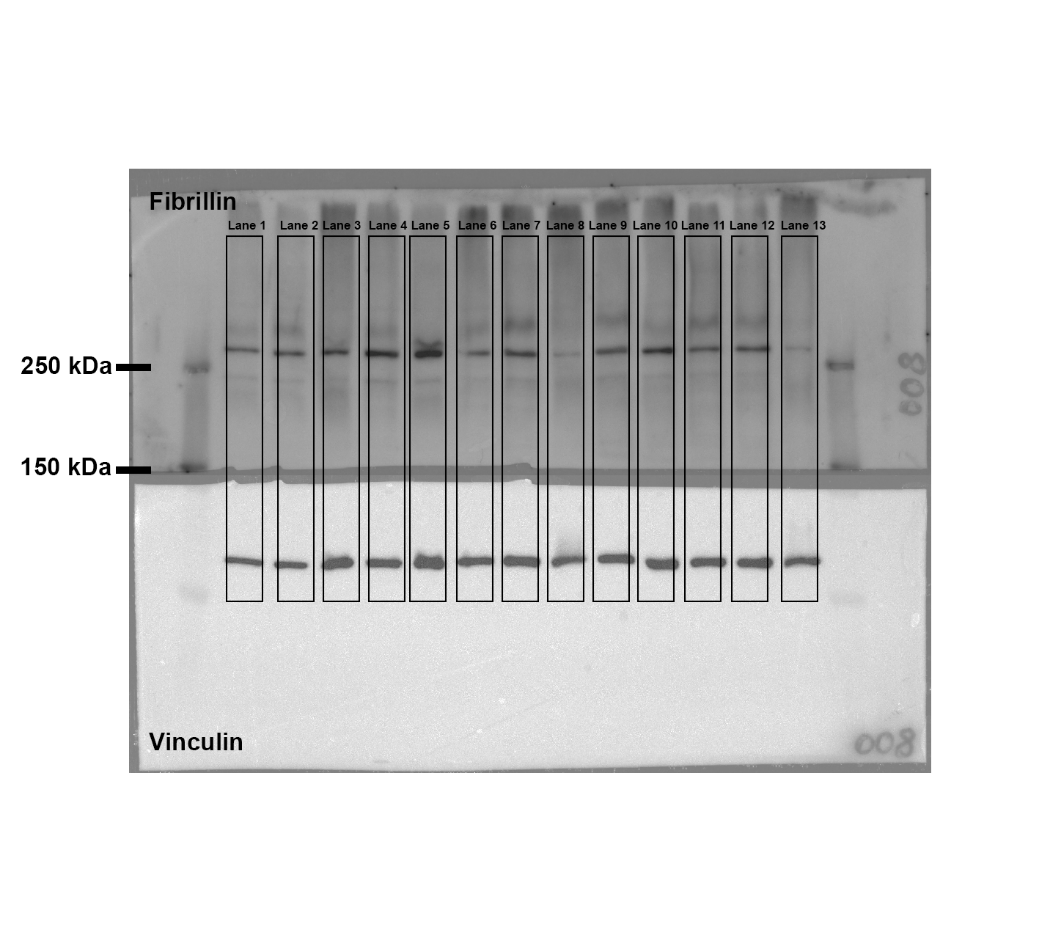


**Figure S4.** Original PVDF membrane for quantification of fibrillin expression. Upper panel: fibrillin, lower panel: vinculin. Lane Lane 1-3: CD; Lane 4, 5: CD-VAD; Lane 6, 7: HFD; Lane 8, 9: HFD-VAD; Lane 10-13: other samples. Membrane was cut at 150 kDa for simultaneous development of target and housekeeper. Chemiluminescence and colorimetry (for marker bands) were merged with the Chemidoc MP imaging system.


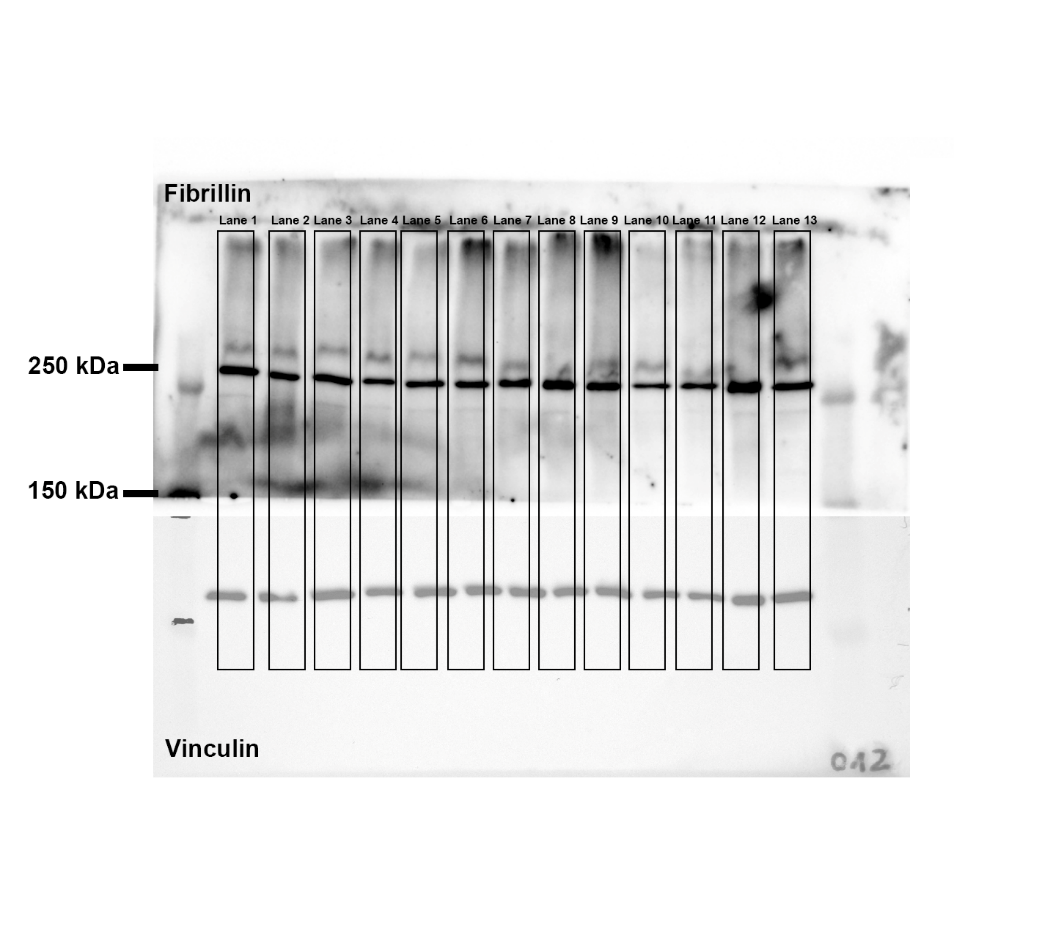


**Figure S5.** Original PVDF membrane for quantification of fibrillin expression. Upper panel: fibrillin, lower panel: vinculin. Lane Lane 1-3: CD; Lane 4, 5: HFD; Lane 6, 7: HFD-VAD; Lane 8-13: other samples. Membrane was cut at 150 kDa for simultaneous development of target and housekeeper. Chemiluminescence and colorimetry (for marker bands) were merged with the Chemidoc MP imaging system.

**
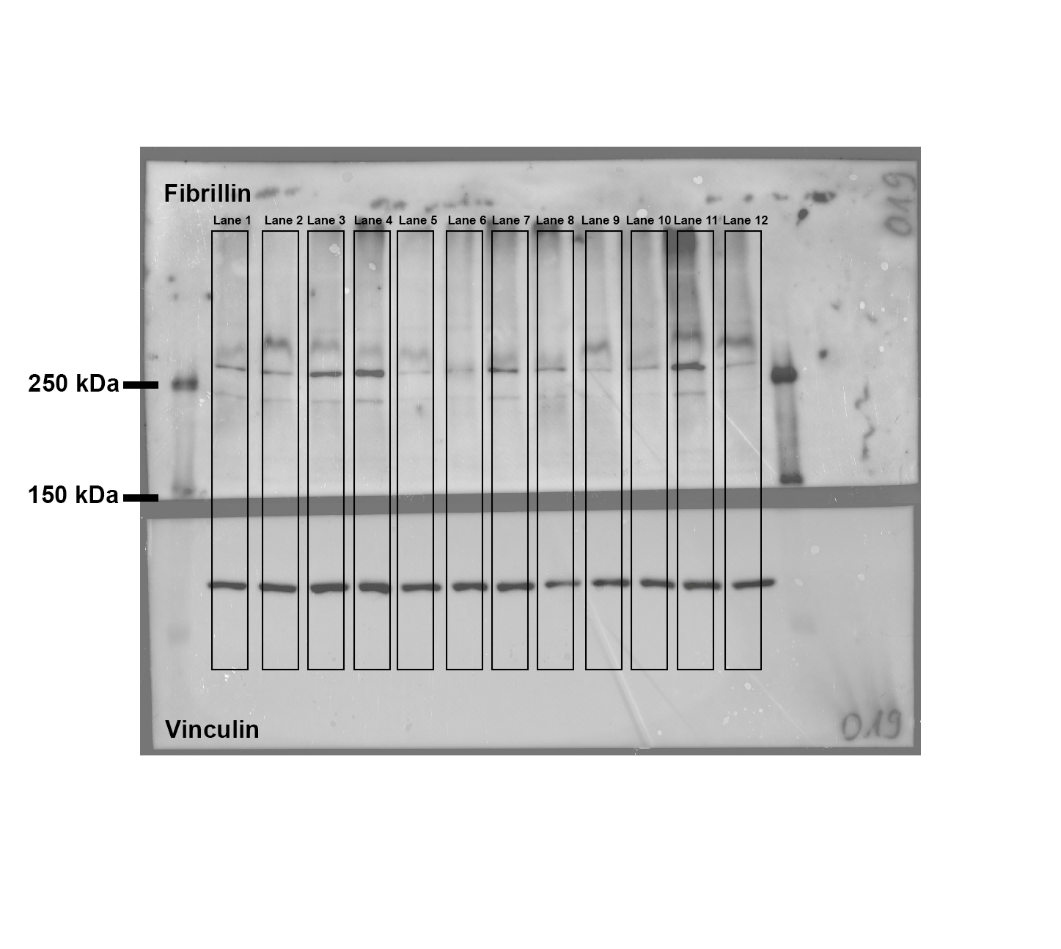
**

**Figure S6.** Original PVDF membrane for quantification of fibrillin expression. Upper panel: fibrillin, lower panel: vinculin. Lane 1, 2: CD; Lane 3, 4: CD-VAD; Lane 5, 6: HFD; Lane 7, 8: HFD-VAD; Lane 9-12: other samples. Membrane was cut at 150 kDa for simultaneous development of target and housekeeper. Chemiluminescence and colorimetry (for marker bands) were merged with the Chemidoc MP imaging system.


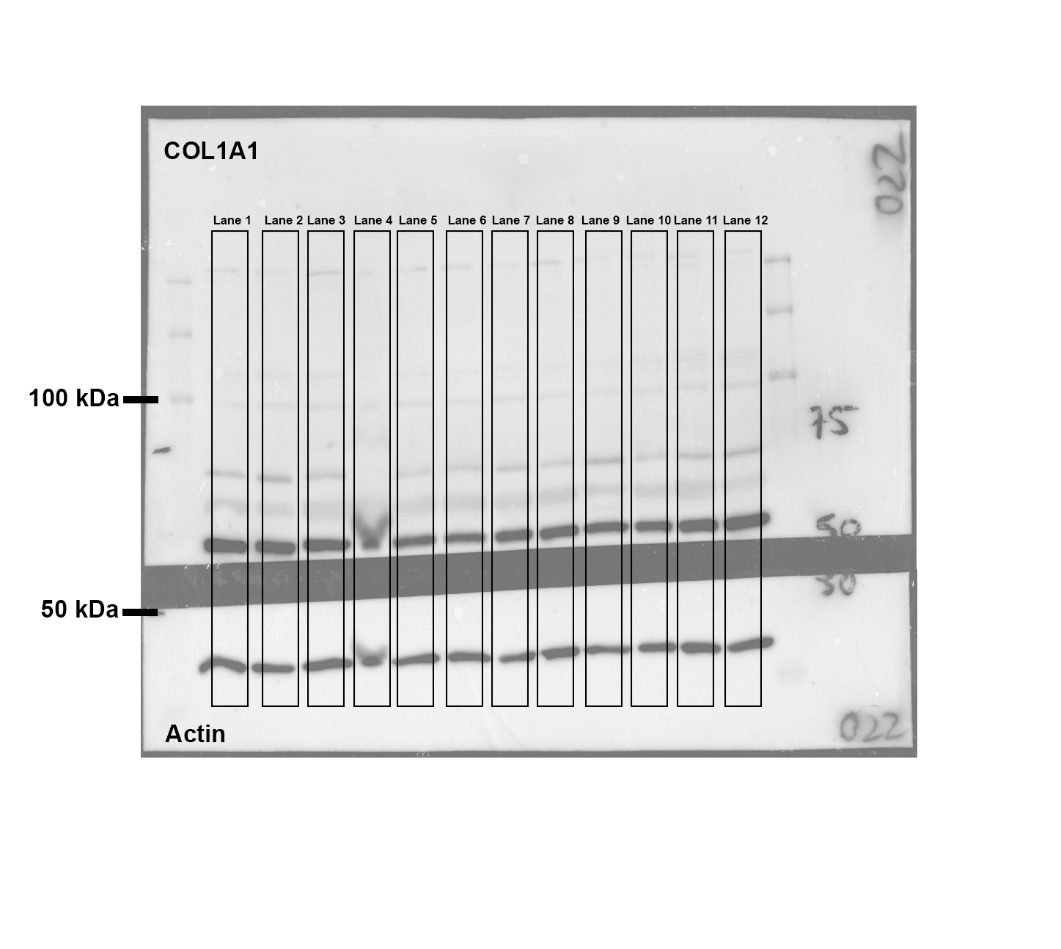


**Figure S7.** Original PVDF membrane for quantification of collagen I expression. Upper panel: COL1A1, lower panel: β-actin. Lane 1, 2: CD; Lane 3, 4: CD-VAD (Lane 4: sample repeated on Fig.S8); Lane 5, 6: HFD; Lane 7, 8: HFD-VAD; Lane 9-12: other samples. Membrane was cut at 50 kDa for simultaneous development of target and housekeeper. Chemiluminescence and colorimetry (for marker bands) were merged with the Chemidoc MP imaging system.


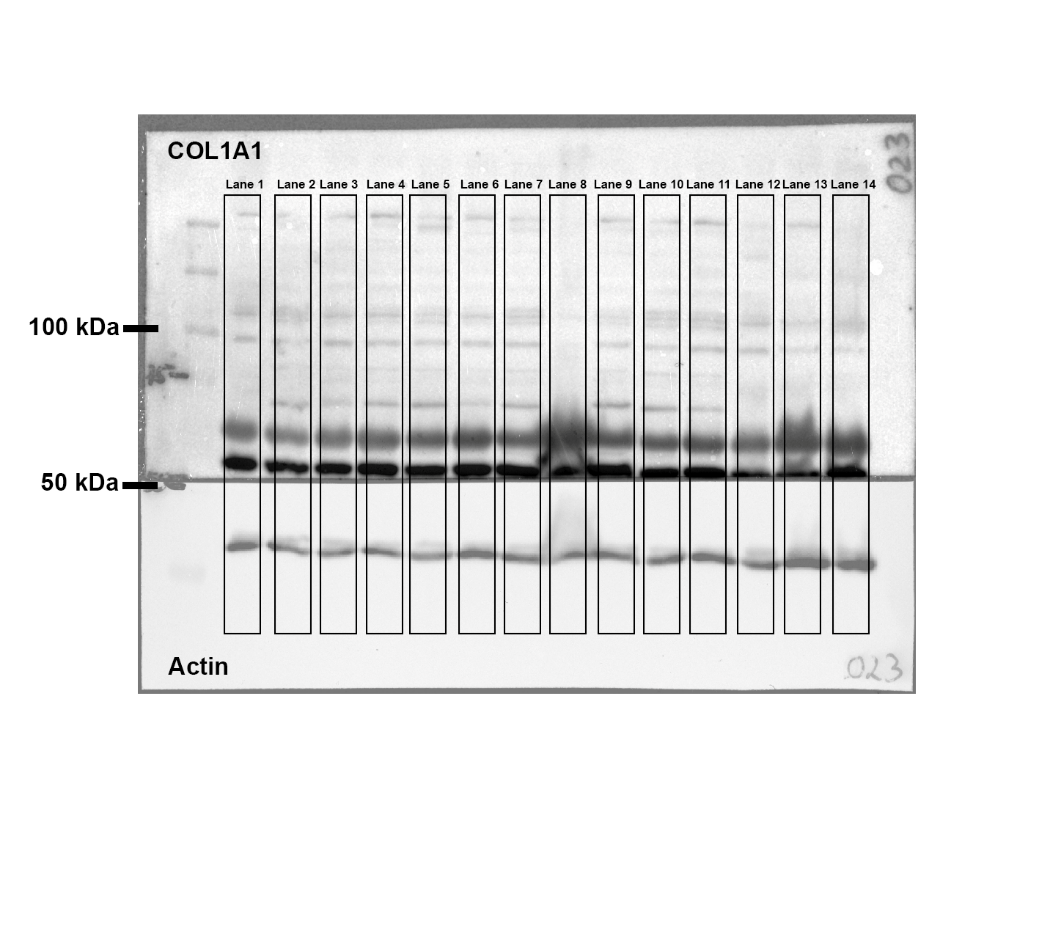


**Figure S8.** Original PVDF membrane for quantification of collagen I expression. Upper panel: COL1A1, lower panel: β-actin. Lane 1, 2: CD; Lane 3-5: CD-VAD; Lane 6, 7: HFD; Lane 8, 9: HFD-VAD); Lane 10-14: other samples. Membrane was cut at 50 kDa for simultaneous development of target and housekeeper. Chemiluminescence and colorimetry (for marker bands) were merged with the Chemidoc MP imaging system.


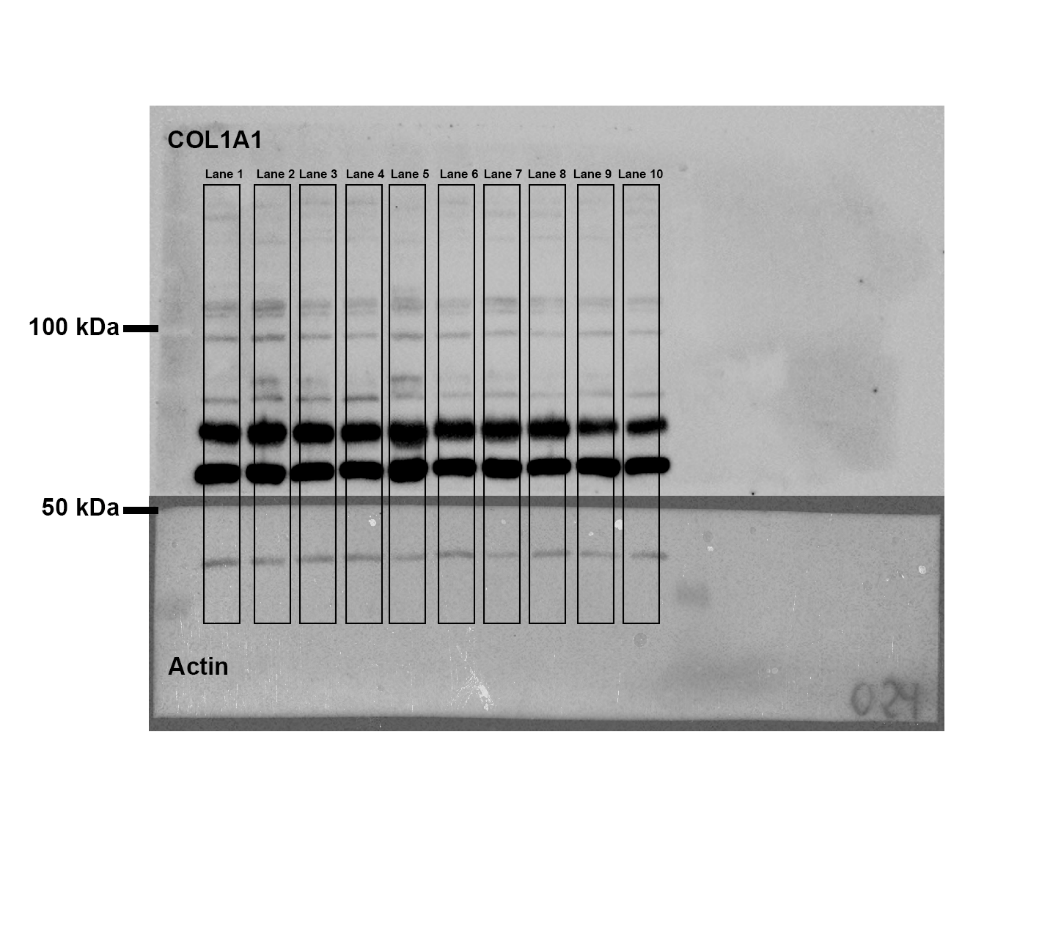


**Figure S9.** Original PVDF membrane for quantification of collagen I expression. Upper panel: COL1A1, lower panel: β-actin. Lane 1, 2: CD; Lane 3, 4: HFD; Lane 5-7: HFD-VAD; Lane 8-10: other samples. Membrane was cut at 50 kDa for simultaneous development of target and housekeeper. Chemiluminescence and colorimetry (for marker bands) were merged with the Chemidoc MP imaging system.


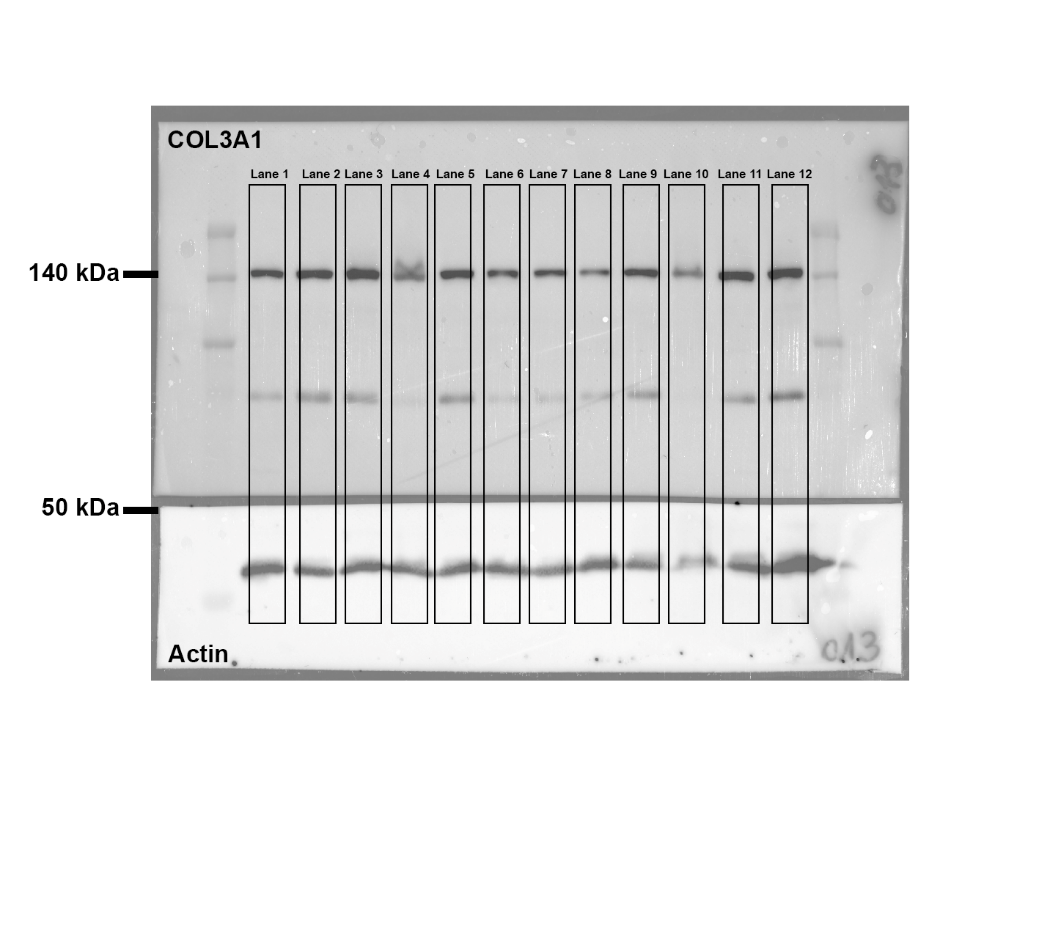


**Figure S10.** Original PVDF membrane for quantification of collagen III expression. Upper panel: COL3A1, lower panel: β-actin. Lane 1, 2: CD; Lane 3, 4: CD-VAD; Lane 5, 6: HFD; Lane:7, 8: HFD-VAD; Lane 9-12: other samples. Membrane was cut at 50 kDa for simultaneous development of target and housekeeper. Chemiluminescence and colorimetry (for marker bands) were merged with the Chemidoc MP imaging system.


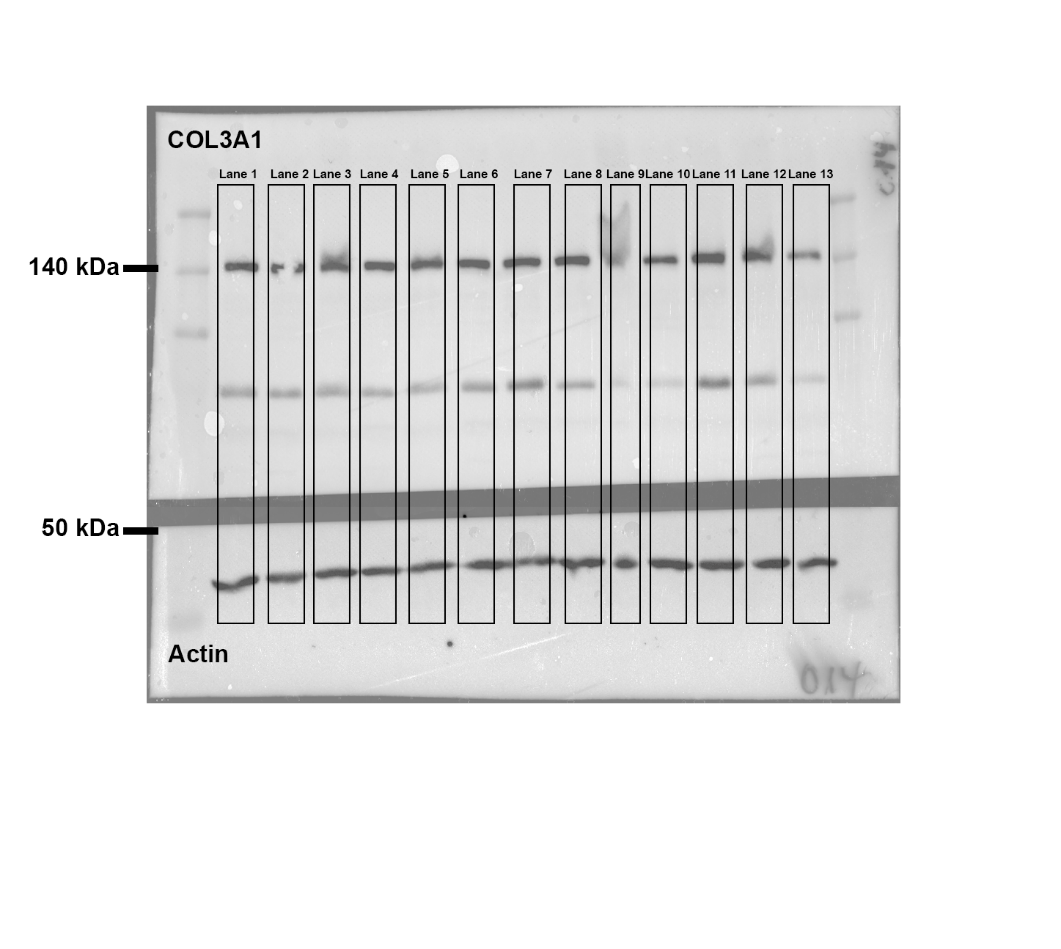


**Figure S11.** Original PVDF membrane for quantification of collagen III expression. Upper panel: COL3A1, lower panel: β-actin. Lane 1, 2: CD; Lane 3, 4: CD-VAD; Lane 5, 6: HFD; Lane:7, 8: HFD-VAD; Lane 9-13: other samples. Membrane was cut at 50 kDa for simultaneous development of target and housekeeper. Chemiluminescence and colorimetry (for marker bands) were merged with the Chemidoc MP imaging system.


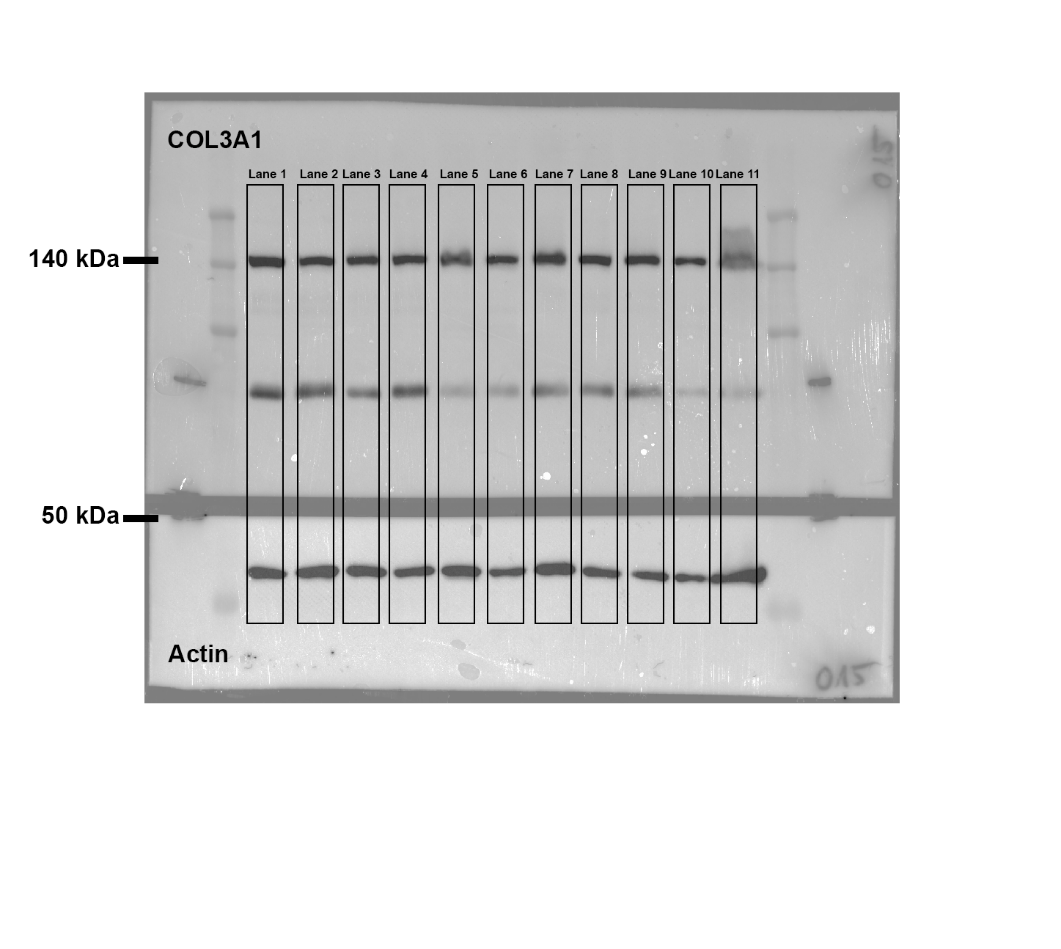


**Figure S12.** Original PVDF membrane for quantification of collagen III expression. Upper panel: COL3A1, lower panel: β-actin. Lane 1-3: CD; Lane 4, 5: HFD; Lane 6, 7: HFD-VAD; Lane: 8-11: other samples. Membrane was cut at 50 kDa for simultaneous development of target and housekeeper. Chemiluminescence and colorimetry (for marker bands) were merged with the Chemidoc MP imaging system.


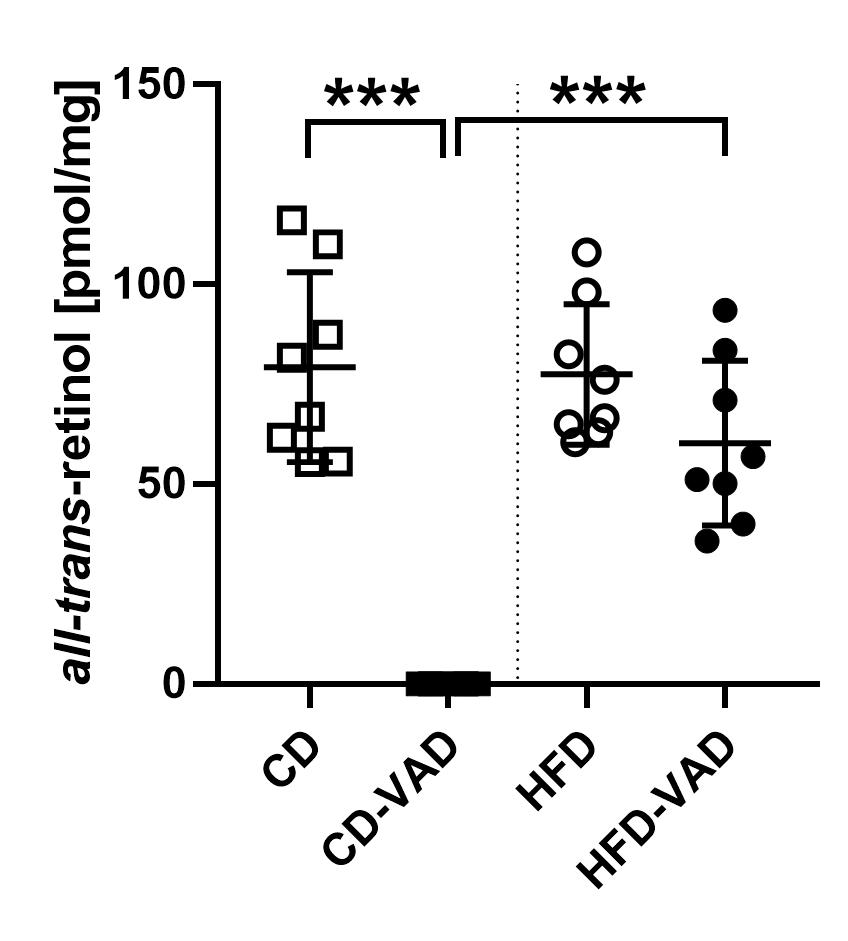


**Figure S13. Adipose tissue concentrations of *all-trans-*retinol.** Data are presented as values of individual mice; group means and SDs are indicated. Statistics: two-Way ANOVA followed by Tukey post hoc analysis; ***p < 0.001. CD, control diet; HFD, high-fat diet; VAD, vitamin A deficiency.
